# Supplementary material for: Real-Time Monitoring of Metabolism during Exercise by Exhaled Breath
Source: Metabolites. 2021 Dec 8;11(12):856. doi: 10.3390/metabo11120856 (PMC8709070; doi:10.3390/metabo11120856)
Supplement: Supplementary file 1 [file metabolites-11-00856-s001.zip › Figure_S1.pdf]

Arterial blood gas

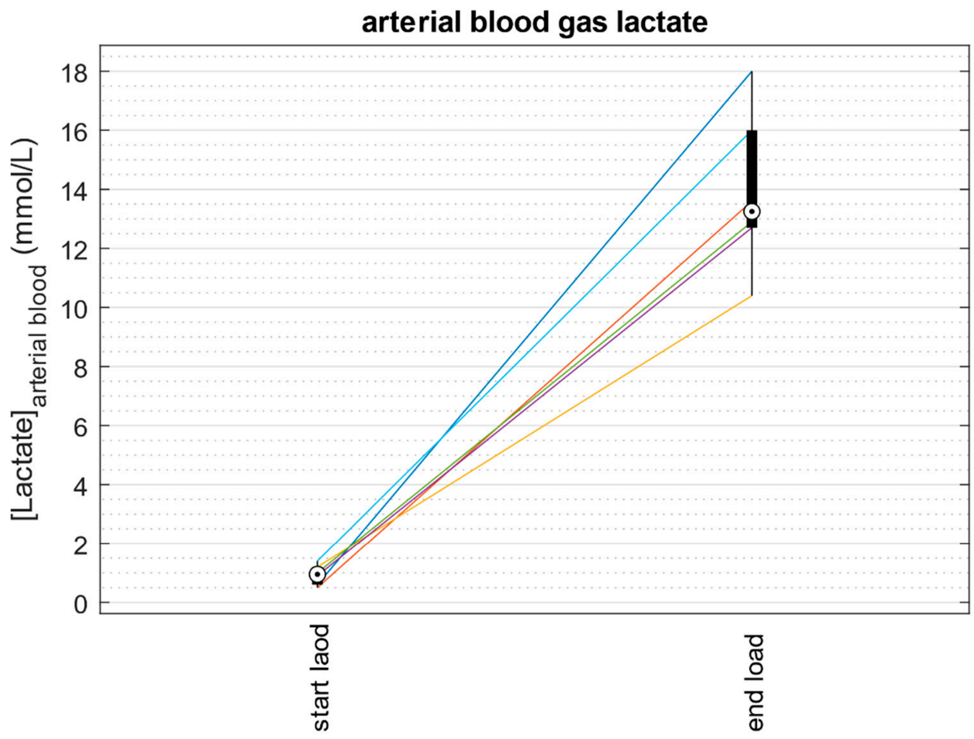

Figure S1. Arterial blood gas measurements of subjects with successful measurements at the beginning of the experiment and at time of complete exhaustion (subject 2 and 7-11). There was a significant increase of arterial blood lactate concentration in all subjects.
