# Supplementary material for: Real-Time Monitoring of Metabolism during Exercise by Exhaled Breath
Source: Metabolites. 2021 Dec 8;11(12):856. doi: 10.3390/metabo11120856 (PMC8709070; doi:10.3390/metabo11120856)
Supplement: Supplementary file 1 [file metabolites-11-00856-s001.zip › Figure_S2.pdf]

Heat maps of features

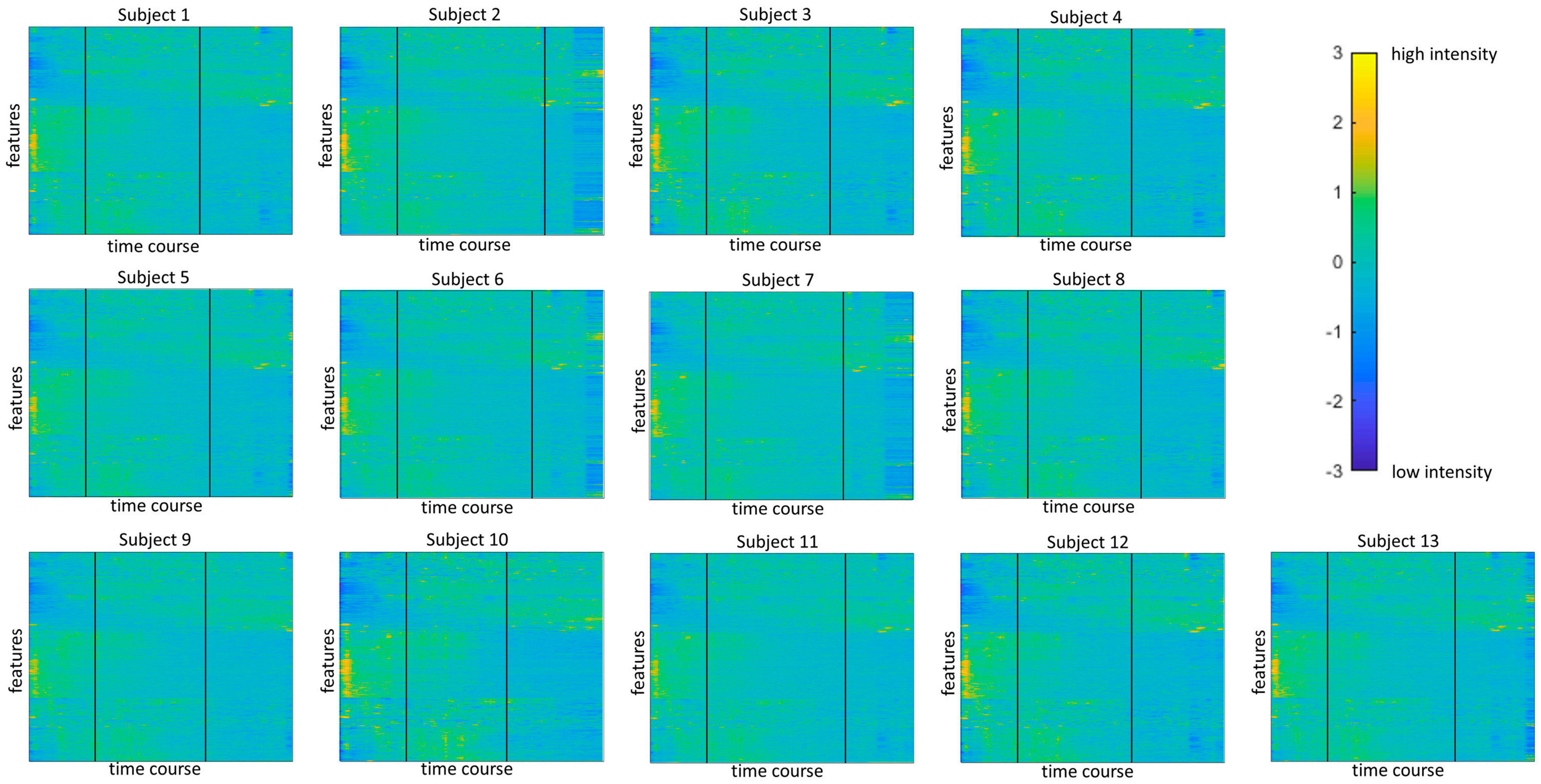

Figure S2. Each heat map represents features over time (x-axis) of one subject (same order of features for all subjects). Black lines are start of the ramp and end of the ramp from left to right. All subjects had similar altering metabolite behaviours during the time course of the exercise protocol. Higher intensities have higher values (up to 3, light yellow), lower intensities have lower values (down to -3, dark blue). The scale of intensities is an arbitrary relative value out of "clusterdata" Matlab algorithm. In clusterdata ward linkage was used and intensities between features were normalized.

Intensities of the mass spectrometric monitoring reflect the Fourier transformation of the image of the current measured at the detector of the orbitrap in arbitrary units – not to be confused with the exercise intensity that reflects the power relative to the athlete.
