# Supplementary material for: Real-Time Monitoring of Metabolism during Exercise by Exhaled Breath
Source: Metabolites. 2021 Dec 8;11(12):856. doi: 10.3390/metabo11120856 (PMC8709070; doi:10.3390/metabo11120856)
Supplement: Supplementary file 1 [file metabolites-11-00856-s001.zip › Figure_S3.pdf]

CPET protocol

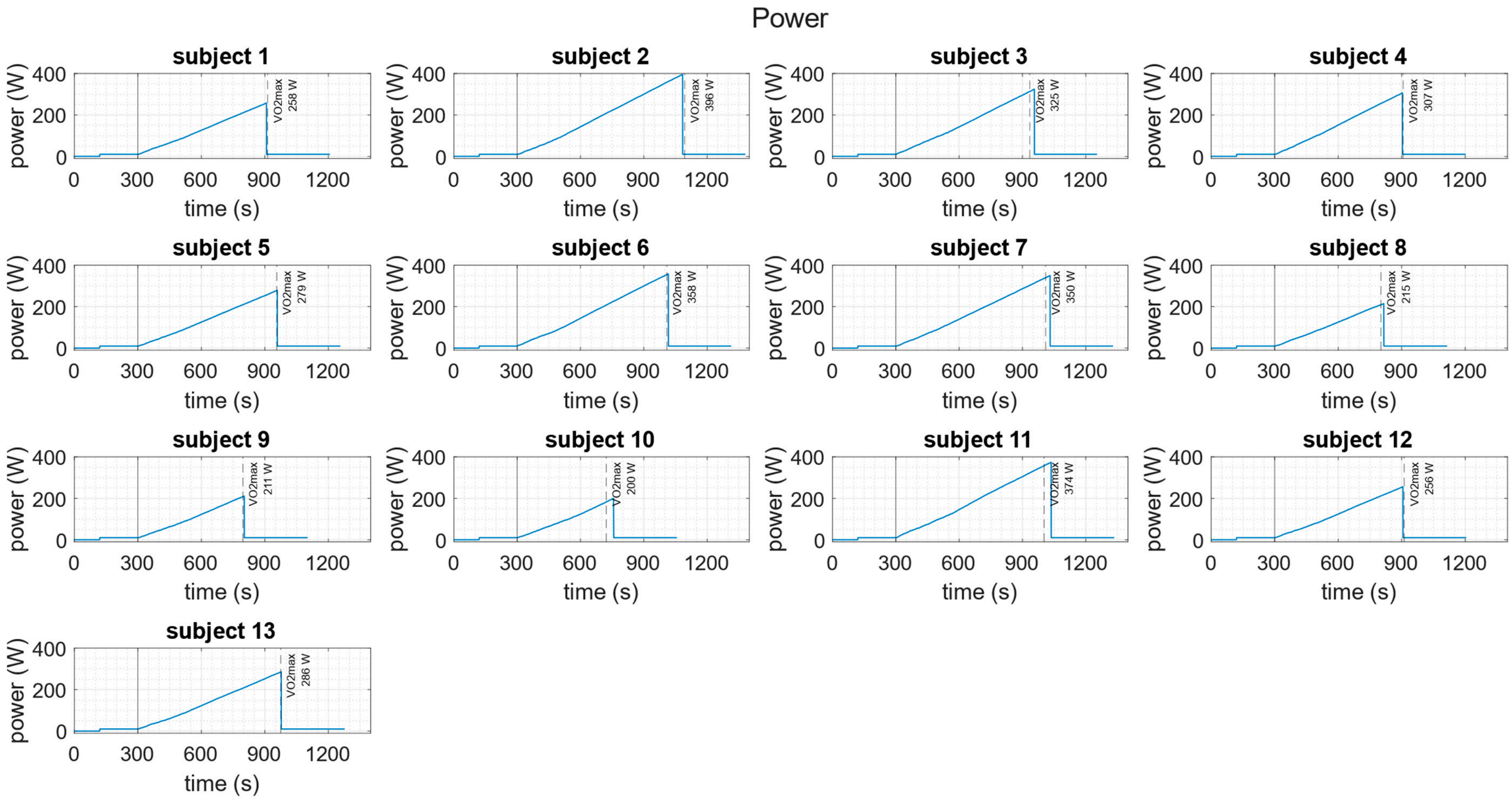

Figure S3. Protocol of each subject. Figures of power over time (blue line). First vertical line is the start of ramp and the second line is the time of the maximal oxygen uptake (VO2max).
