# Supplementary material for: Real-Time Monitoring of Metabolism during Exercise by Exhaled Breath
Source: Metabolites. 2021 Dec 8;11(12):856. doi: 10.3390/metabo11120856 (PMC8709070; doi:10.3390/metabo11120856)

Metabolite intensity for each subject

Figure S4. Figures of intensity over time of all metabolites in the map, with one plot of each subject

Intensities of the mass spectrometric monitoring reflect the Fourier transformation of the image of the current measured at the detector of the orbitrap in arbitrary units – not to be confused with the exercise intensity that reflects the power relative to the athlete.

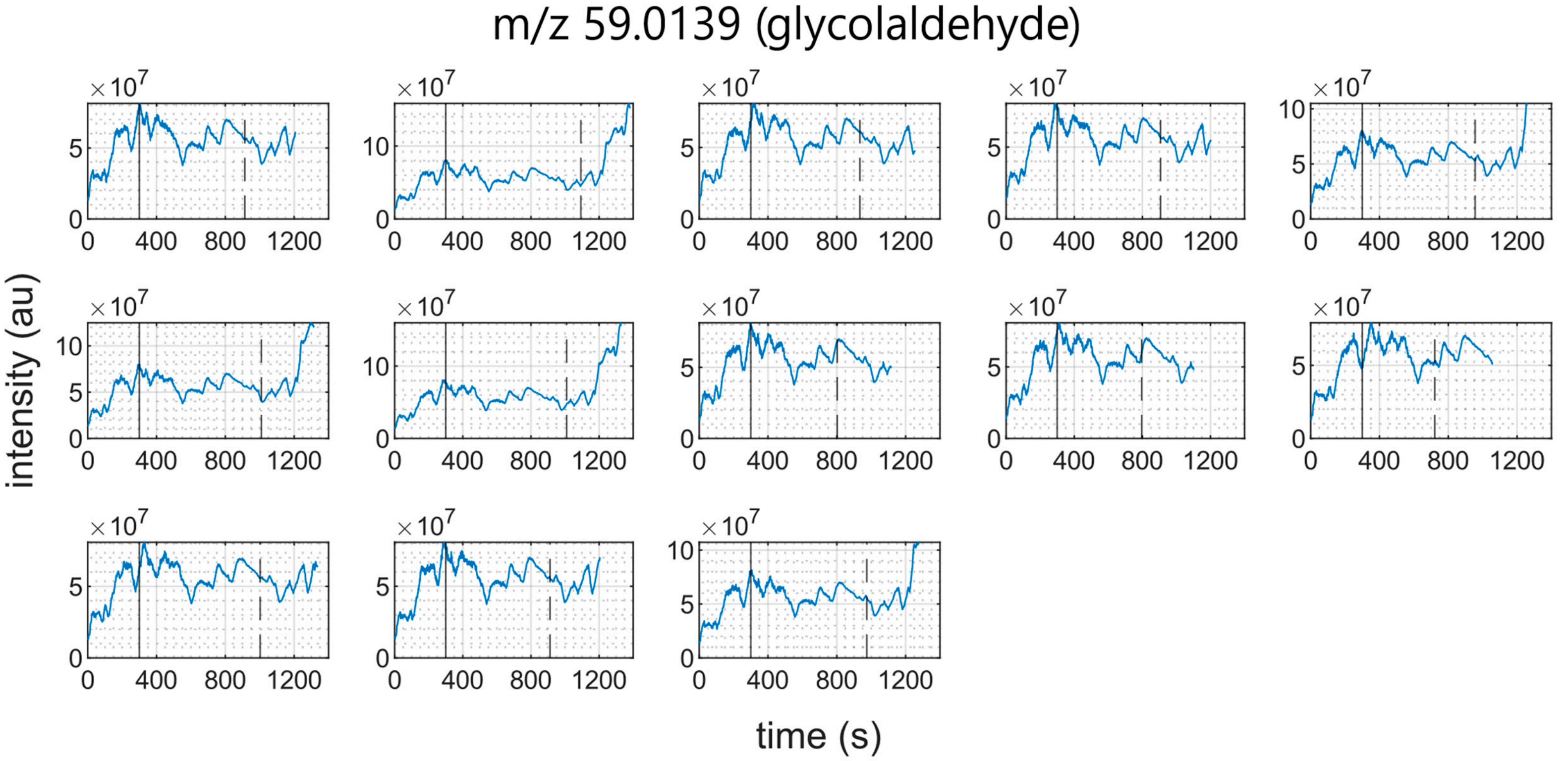

m/z 59.0139 (glycolaldehyde)
intensity (au)
time (s)

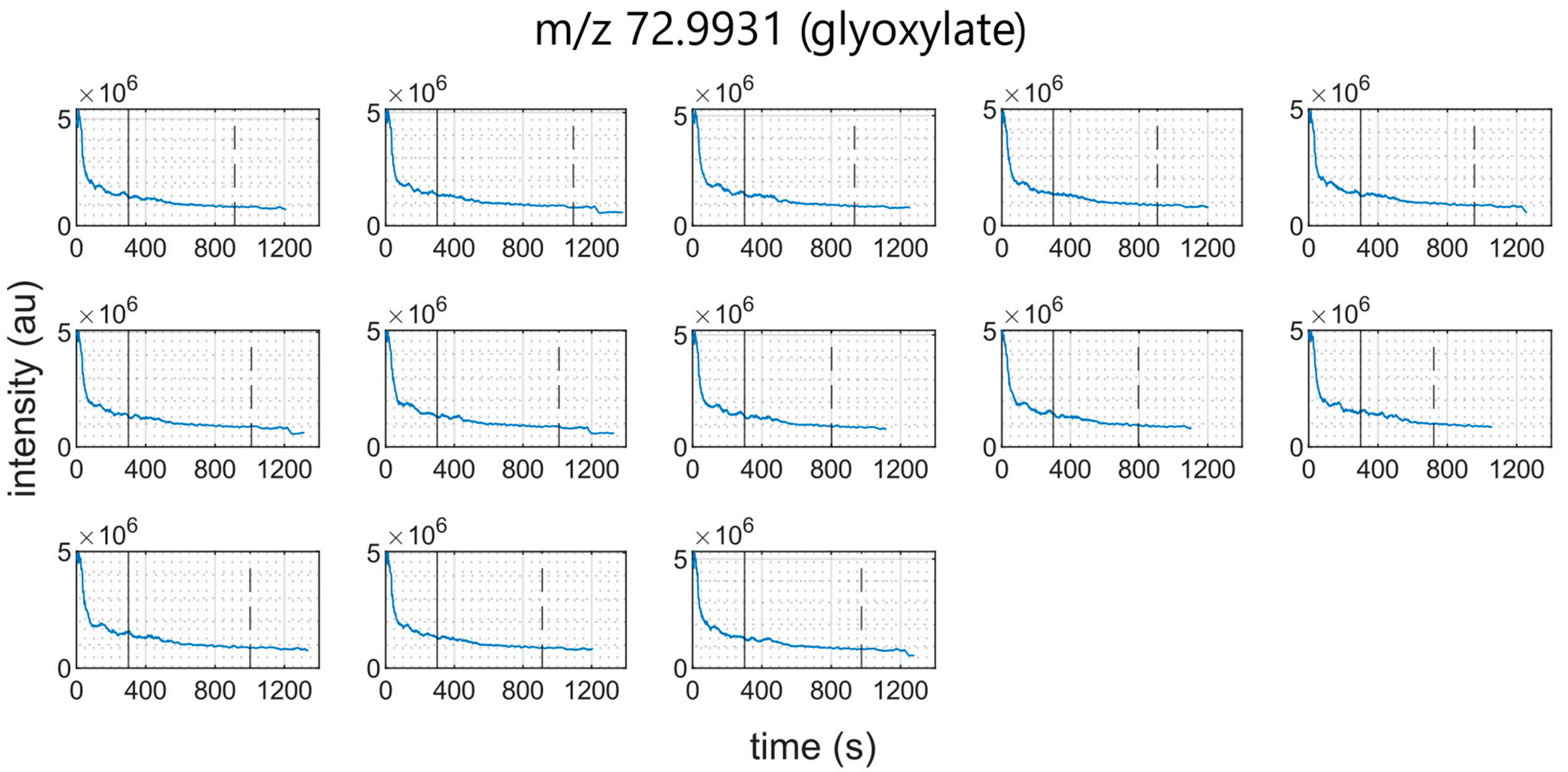
m/z 72.9931 (glyoxylate)
intensity (au)
time (s)
×10^6
5
0
0
400
800
1200

m/z 74.0248 (glycine)

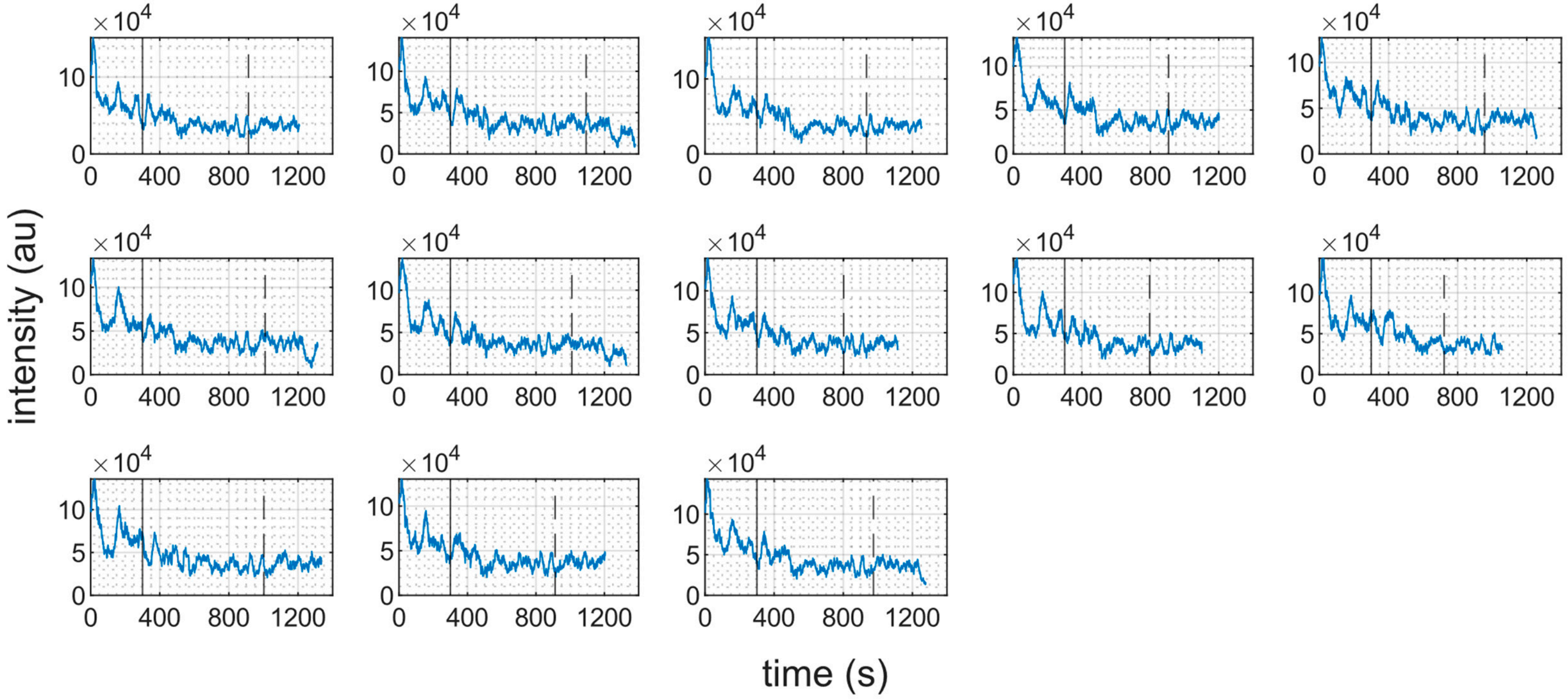

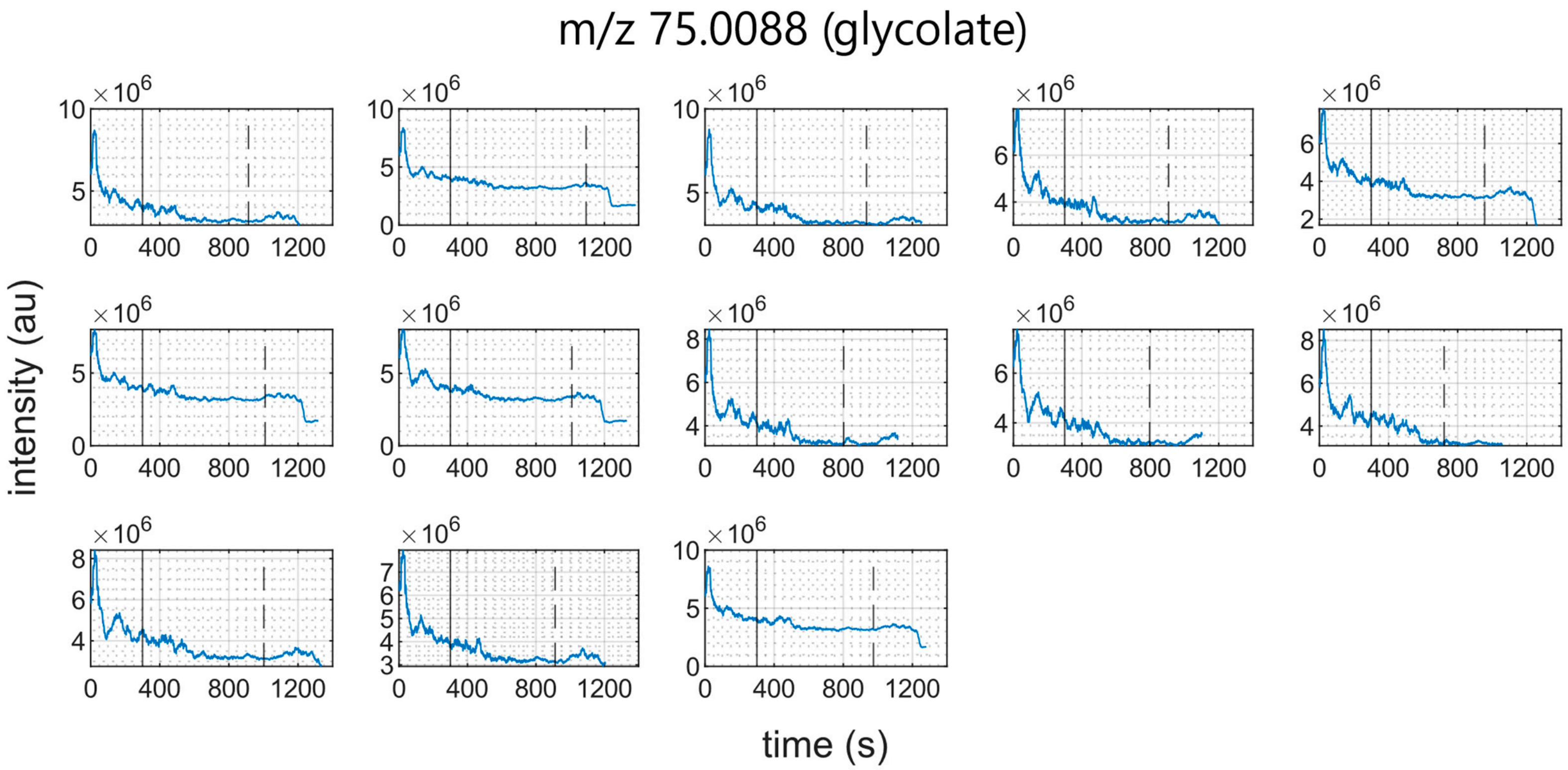

m/z 75.0088 (glycolate)
intensity (au)
time (s)
×10^6
0
400
800
1200

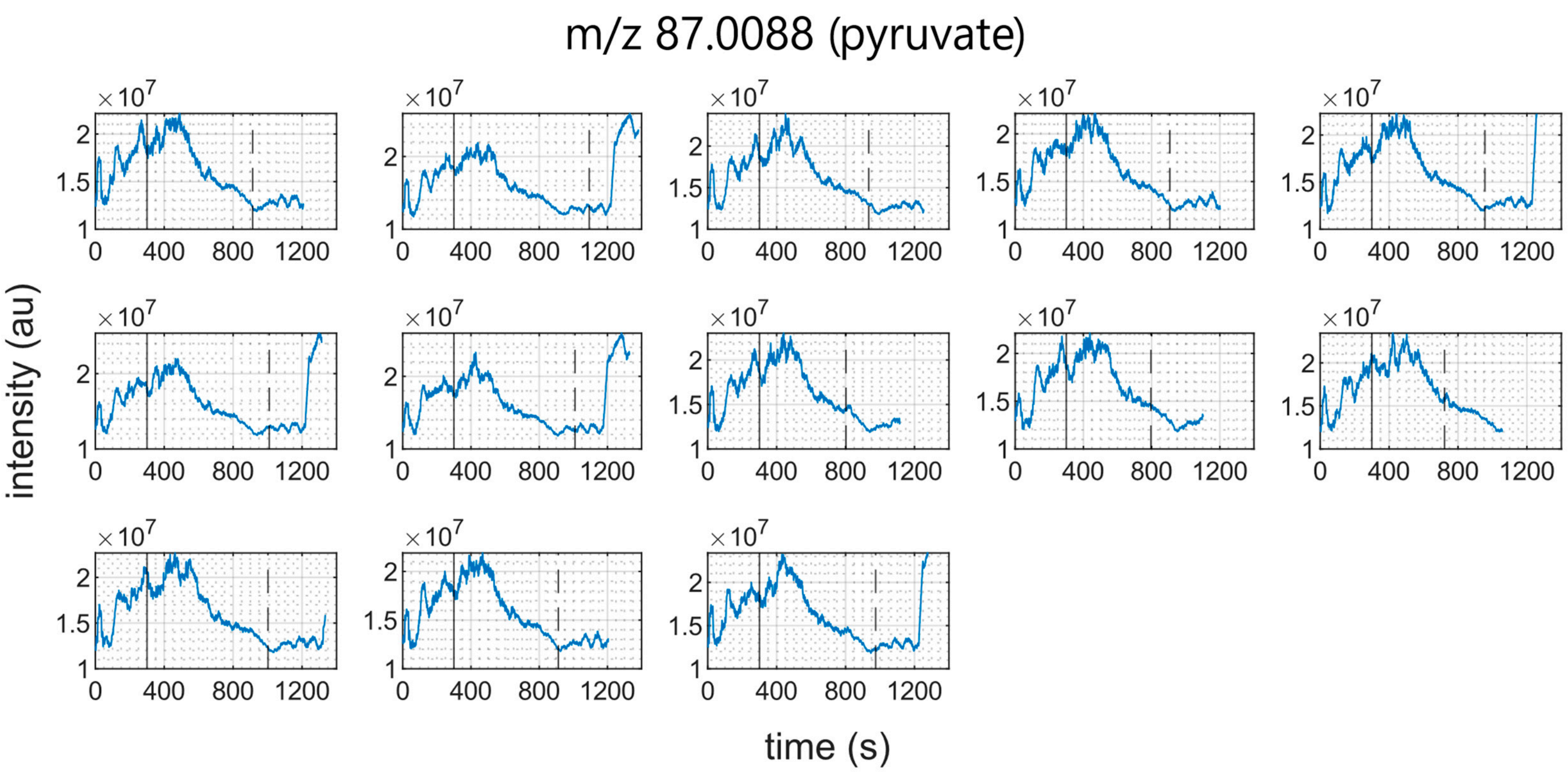
m/z 87.0088 (pyruvate)
×10^7
intensity (au)
time (s)
0 400 800 1200
1 1.5 2

m/z 88.9881 (oxalate)

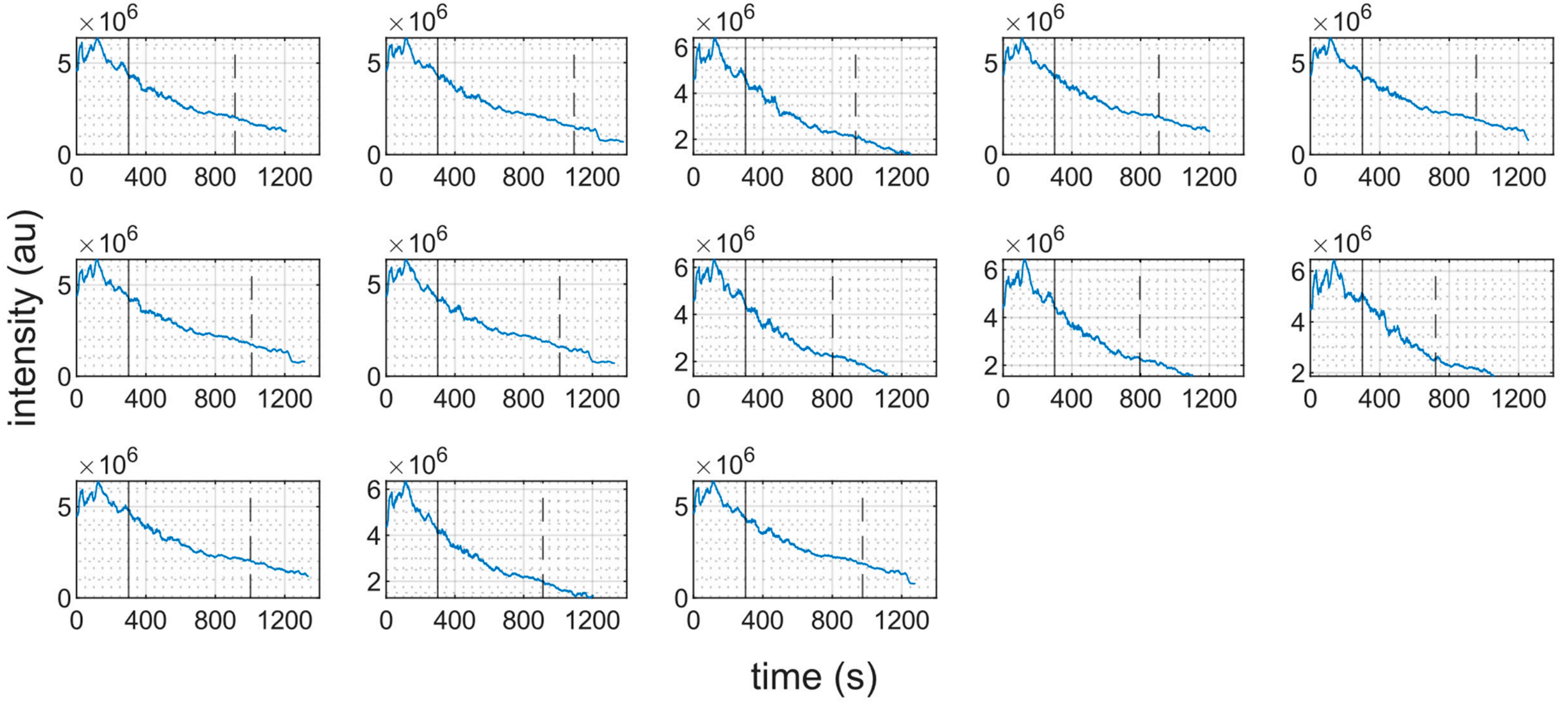

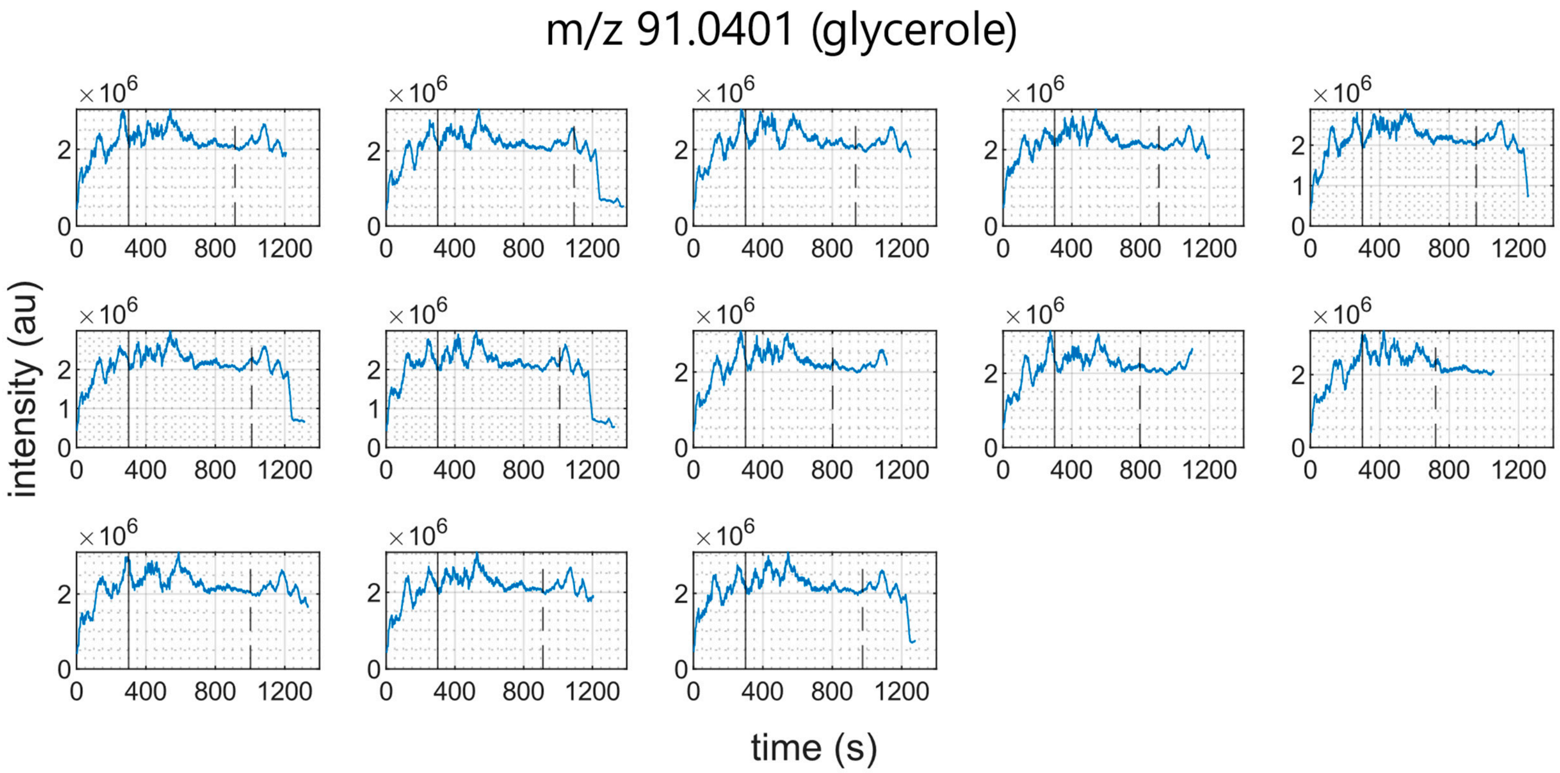

m/z 91.0401 (glycerole)
×10^6
intensity (au)
time (s)
0
400
800
1200
0
1
2

m/z 101.0244
(acetoacetate/oxobutyric acid)

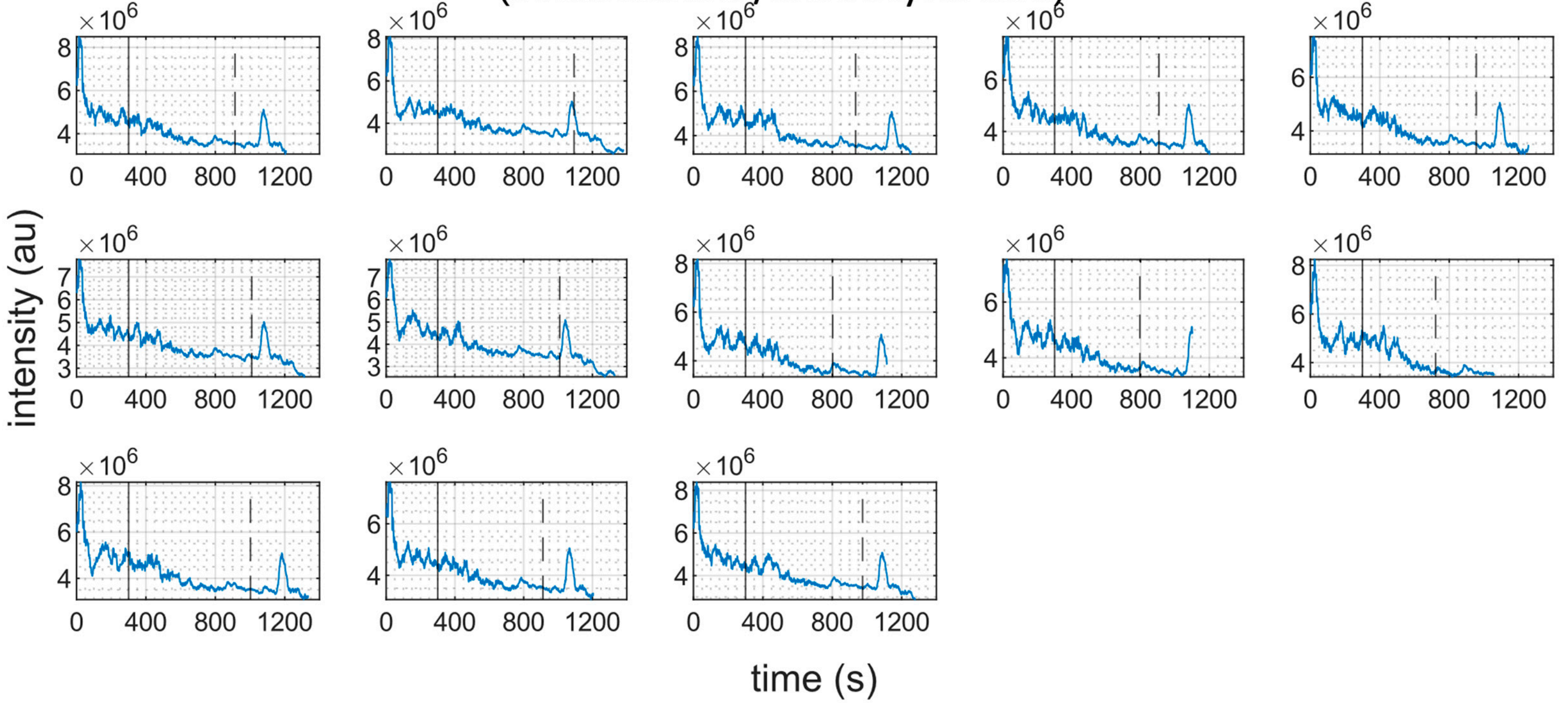

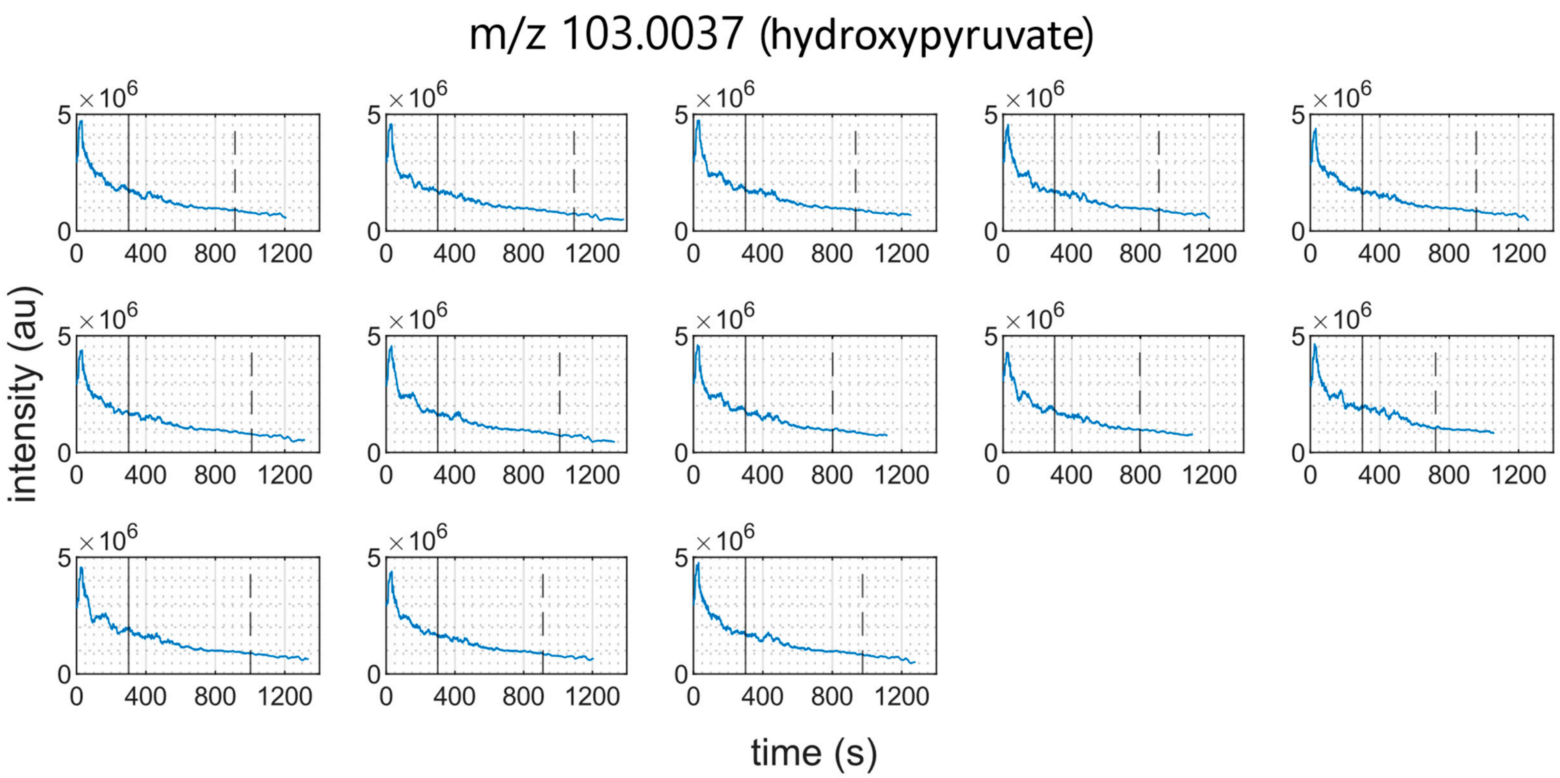

m/z 103.0037 (hydroxypyruvate)
intensity (au)
time (s)
×10^6
5
0
0
400
800
1200

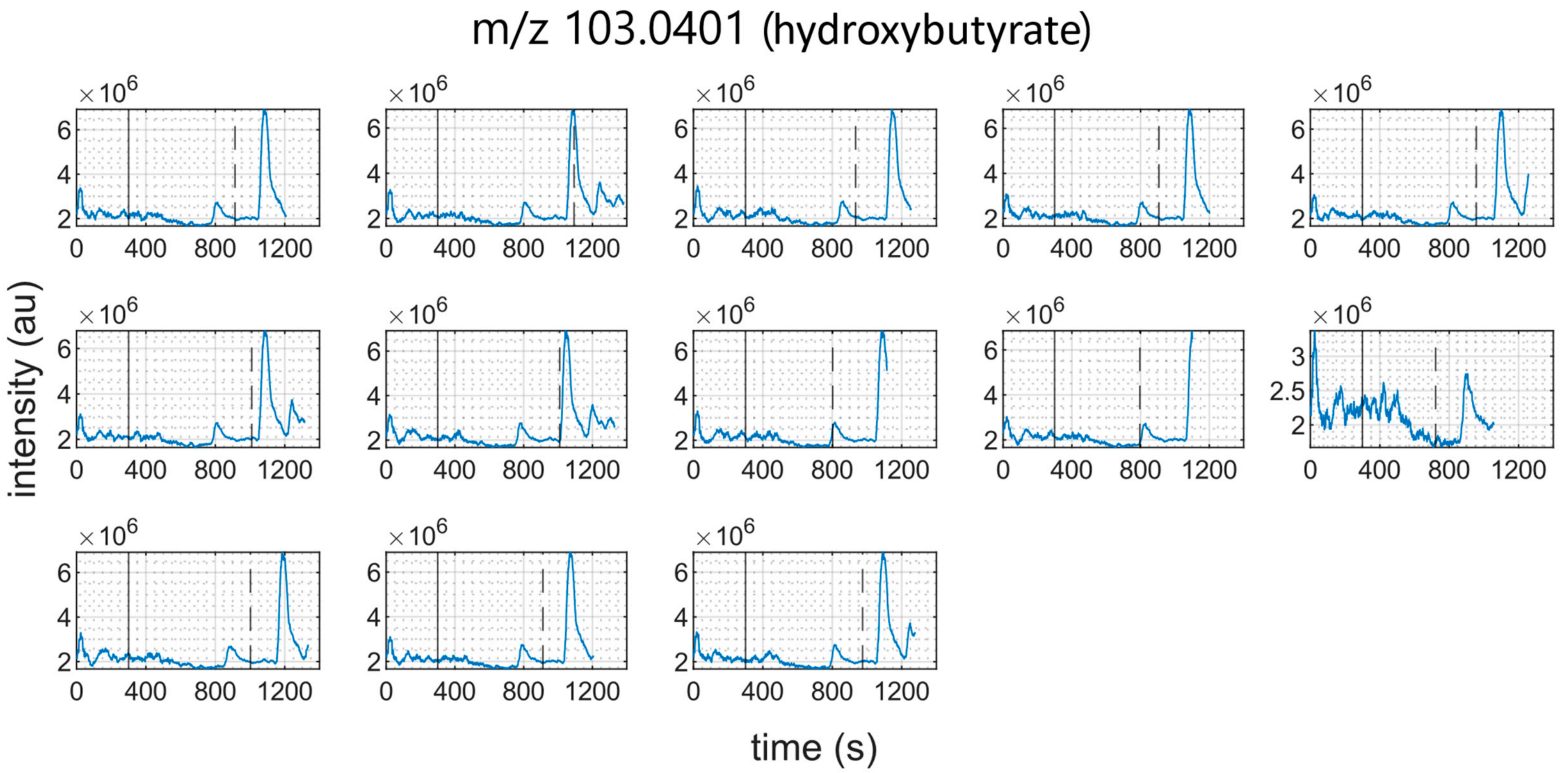
m/z 103.0401 (hydroxybutyrate)
intensity (au)
time (s)
×10^6

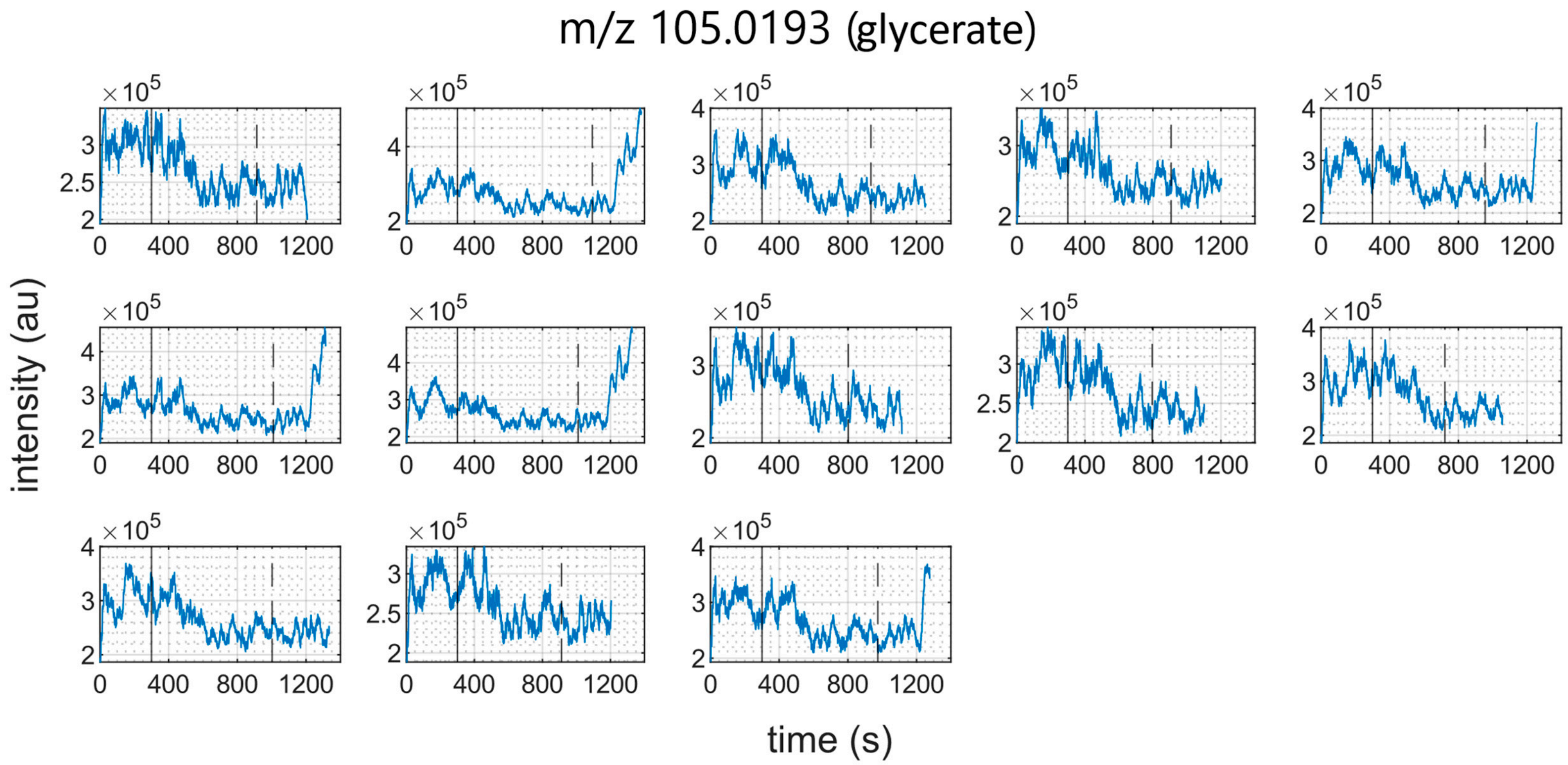

m/z 105.0193 (glycerate)
intensity (au)
time (s)
×10^5

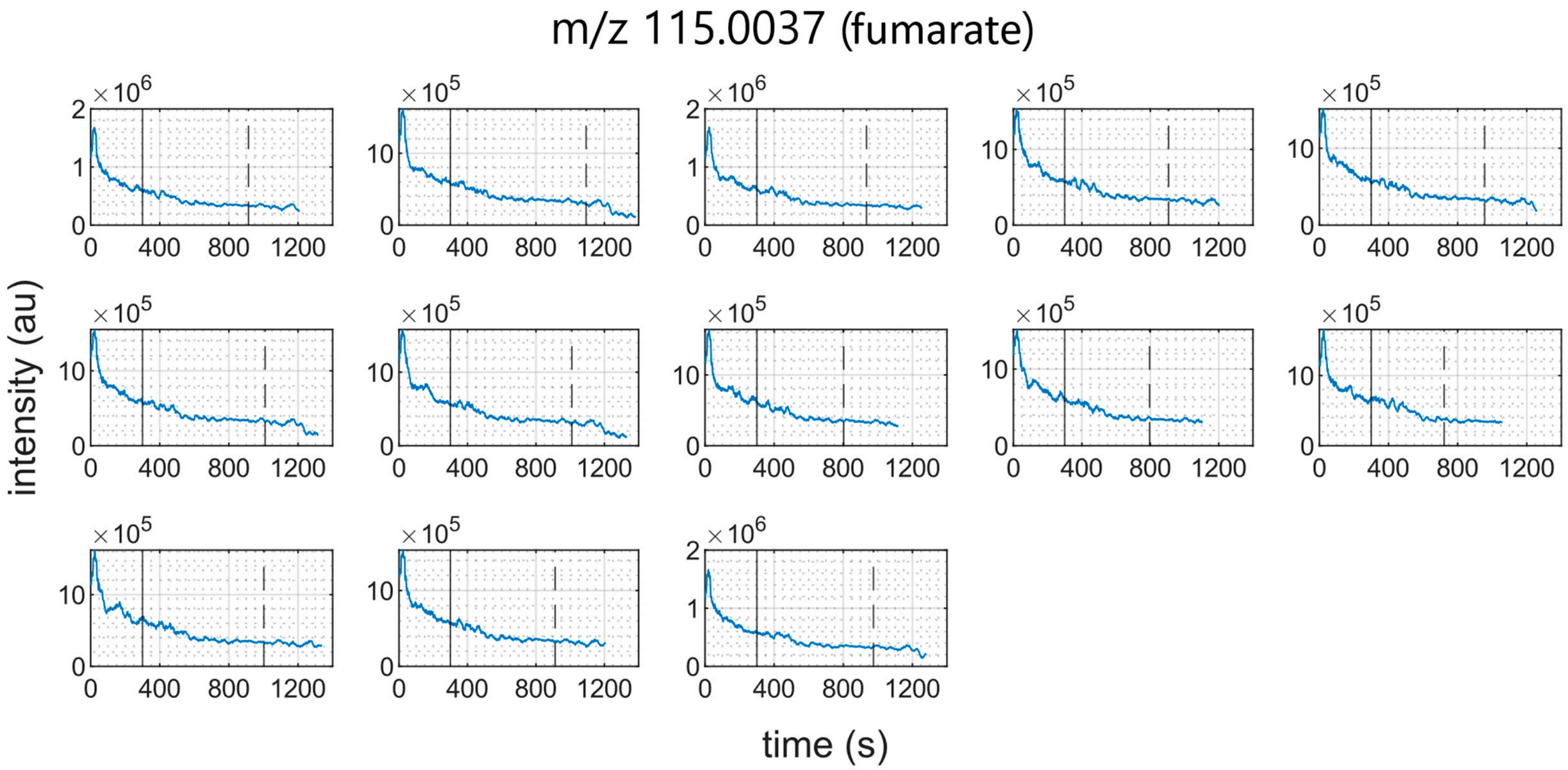
m/z 115.0037 (fumarate)
intensity (au)
time (s)

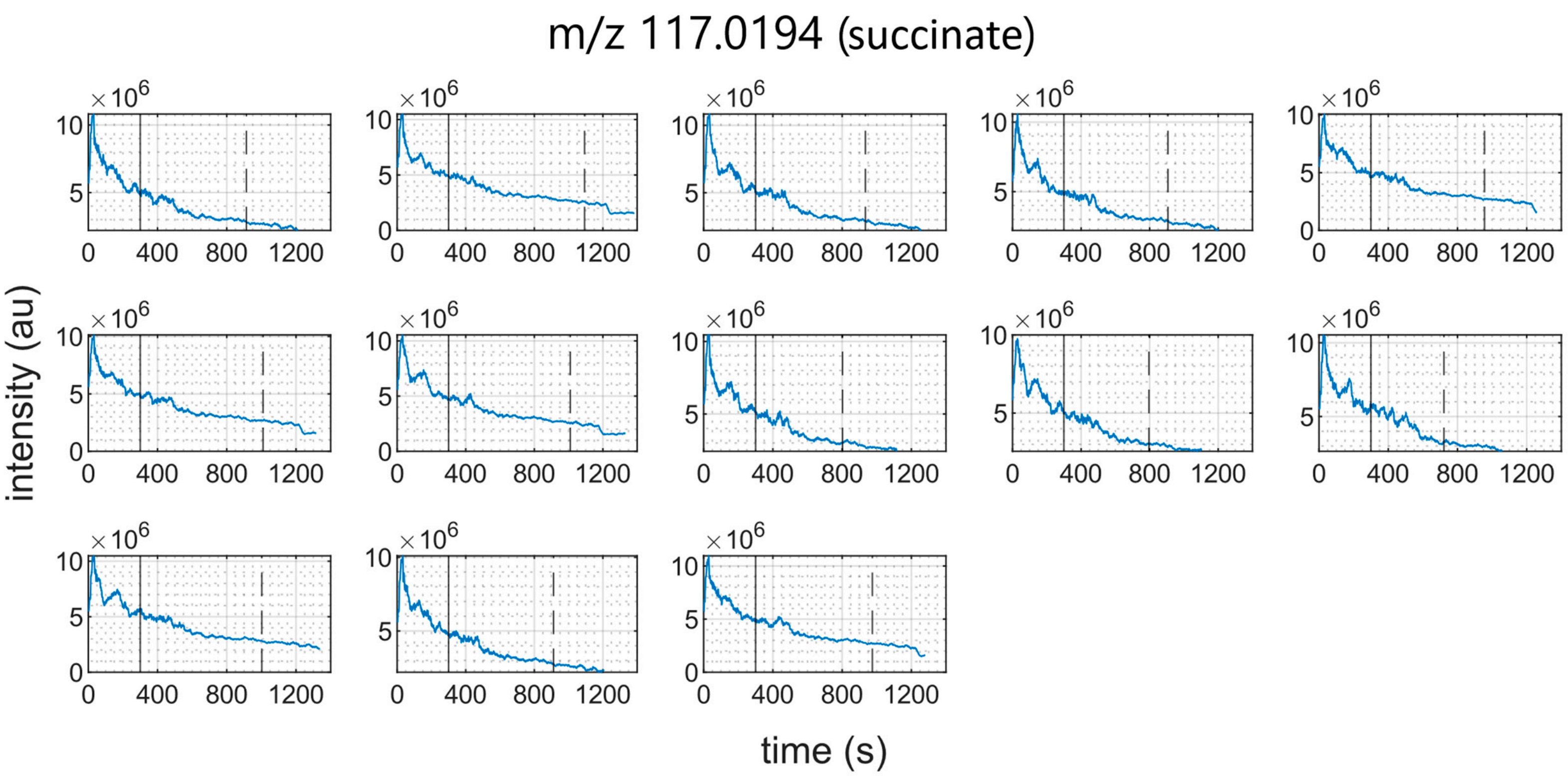
m/z 117.0194 (succinate)
intensity (au)
time (s)
×10^6
0
400
800
1200
10
5
0

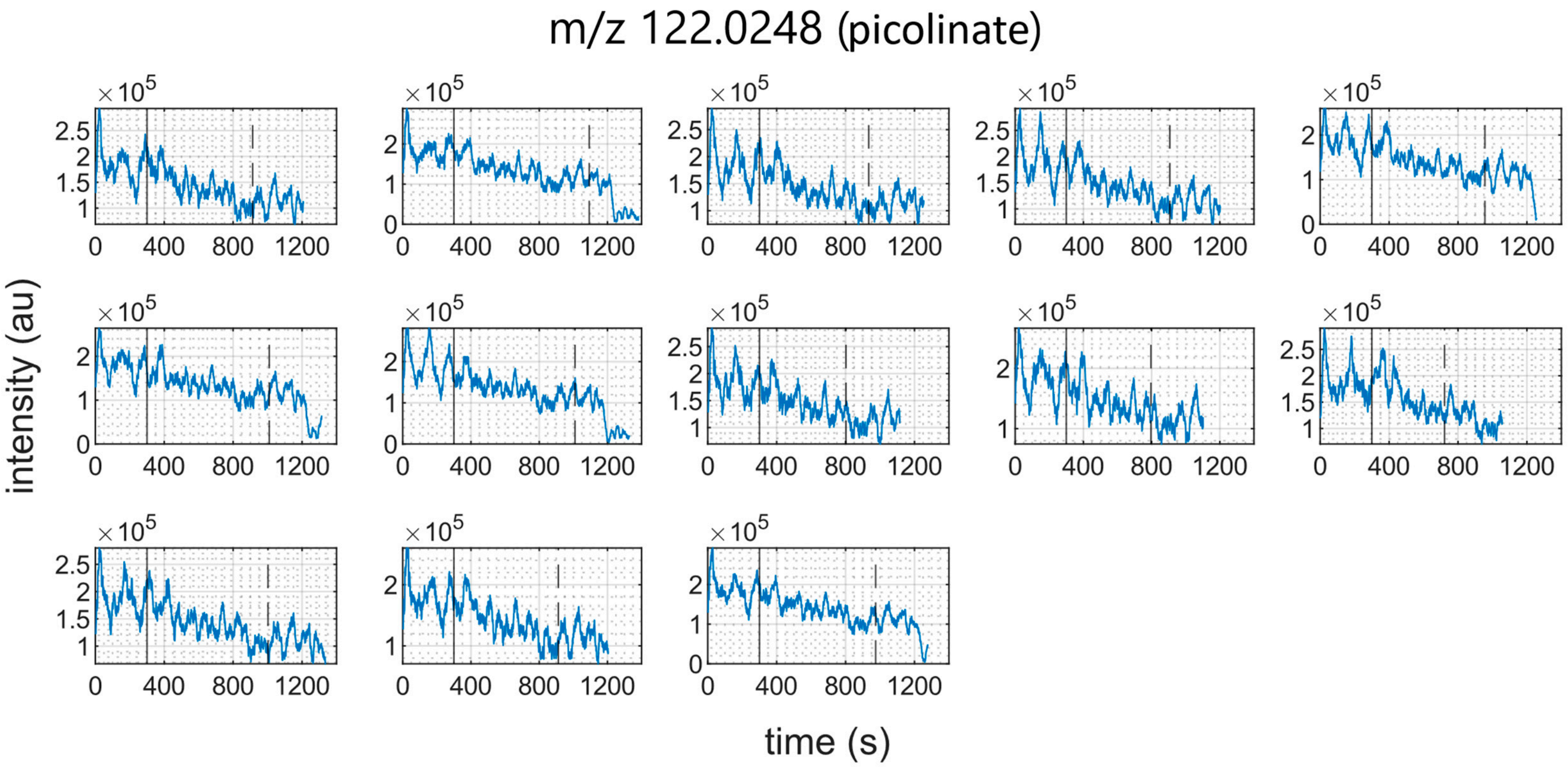
m/z 122.0248 (picolinate)
intensity (au)
time (s)
×10^5
0
400
800
1200

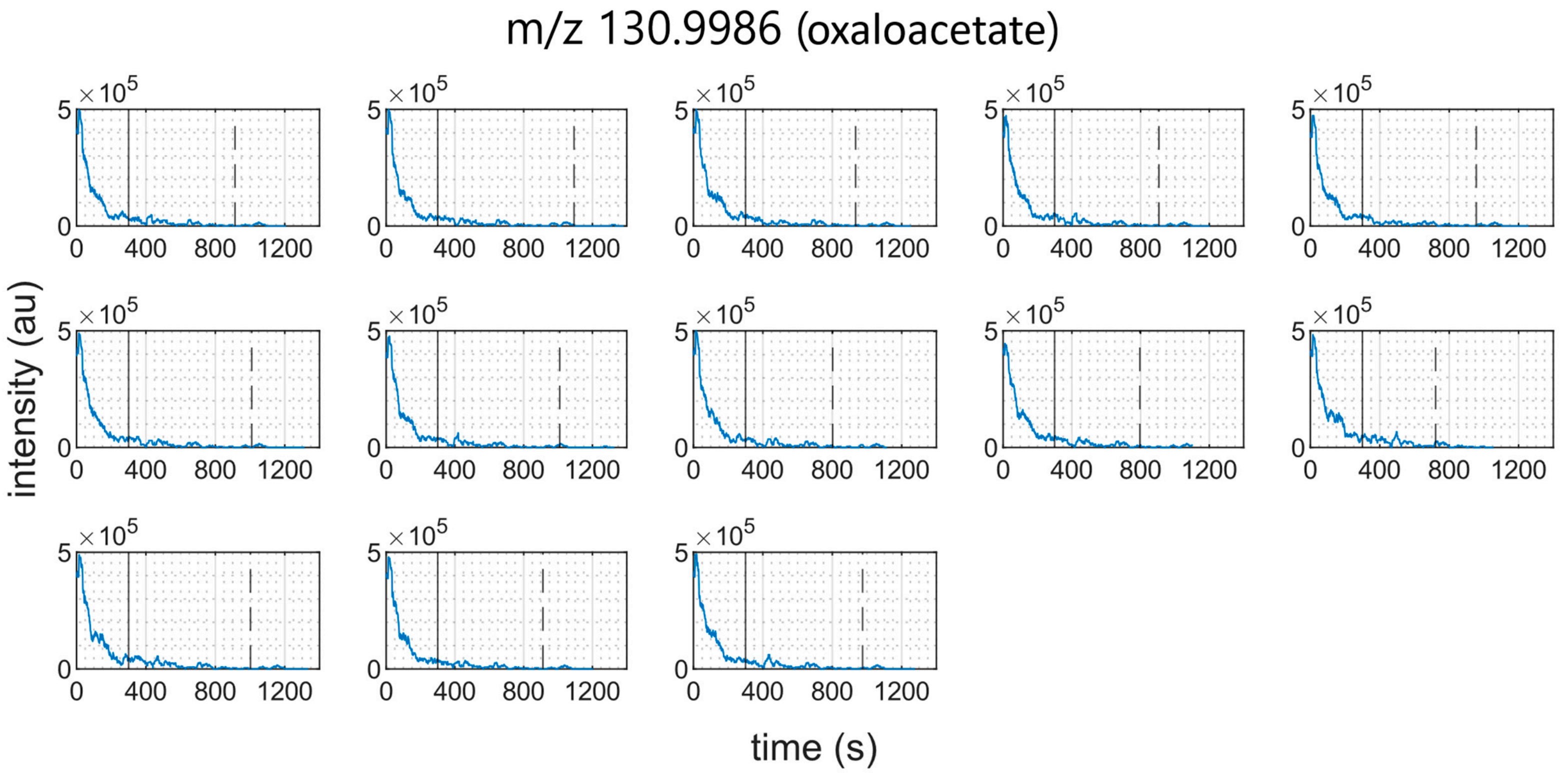
m/z 130.9986 (oxaloacetate)
×10^5
5
0
0
400
800
1200
intensity (au)
time (s)

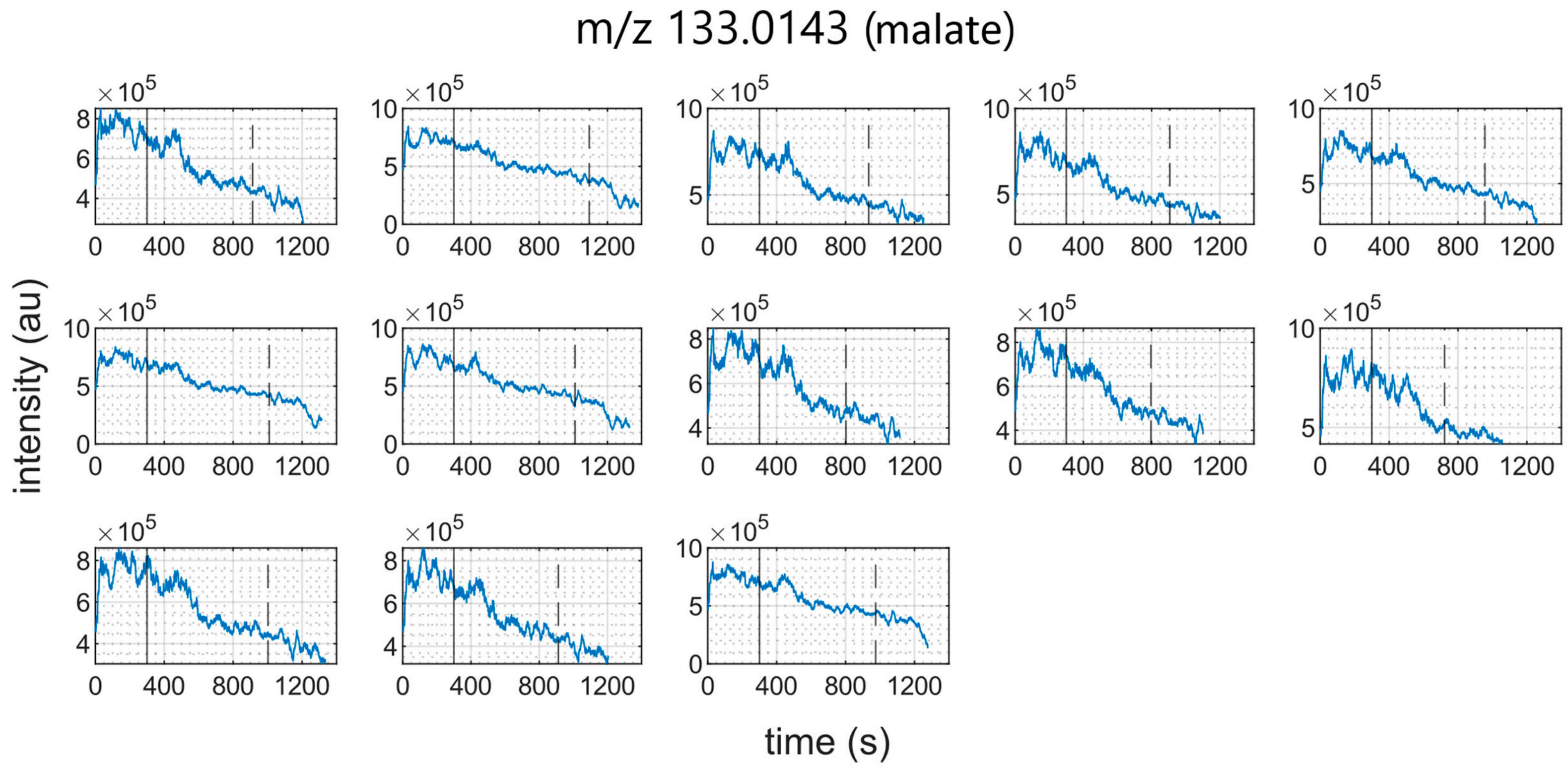
m/z 133.0143 (malate)
intensity (au)
time (s)
×10^5
0
400
800
1200

m/z 133.0507 (deoxypentose)

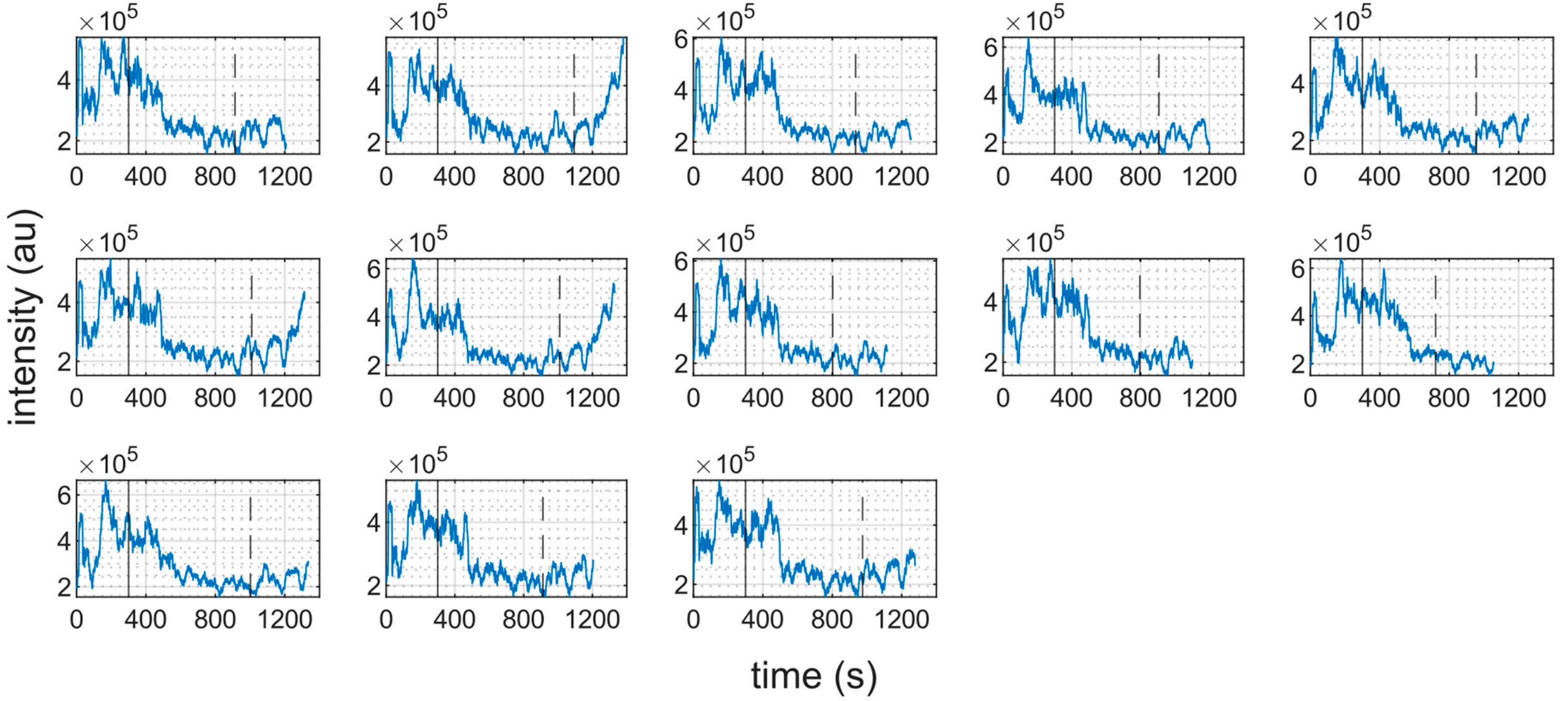

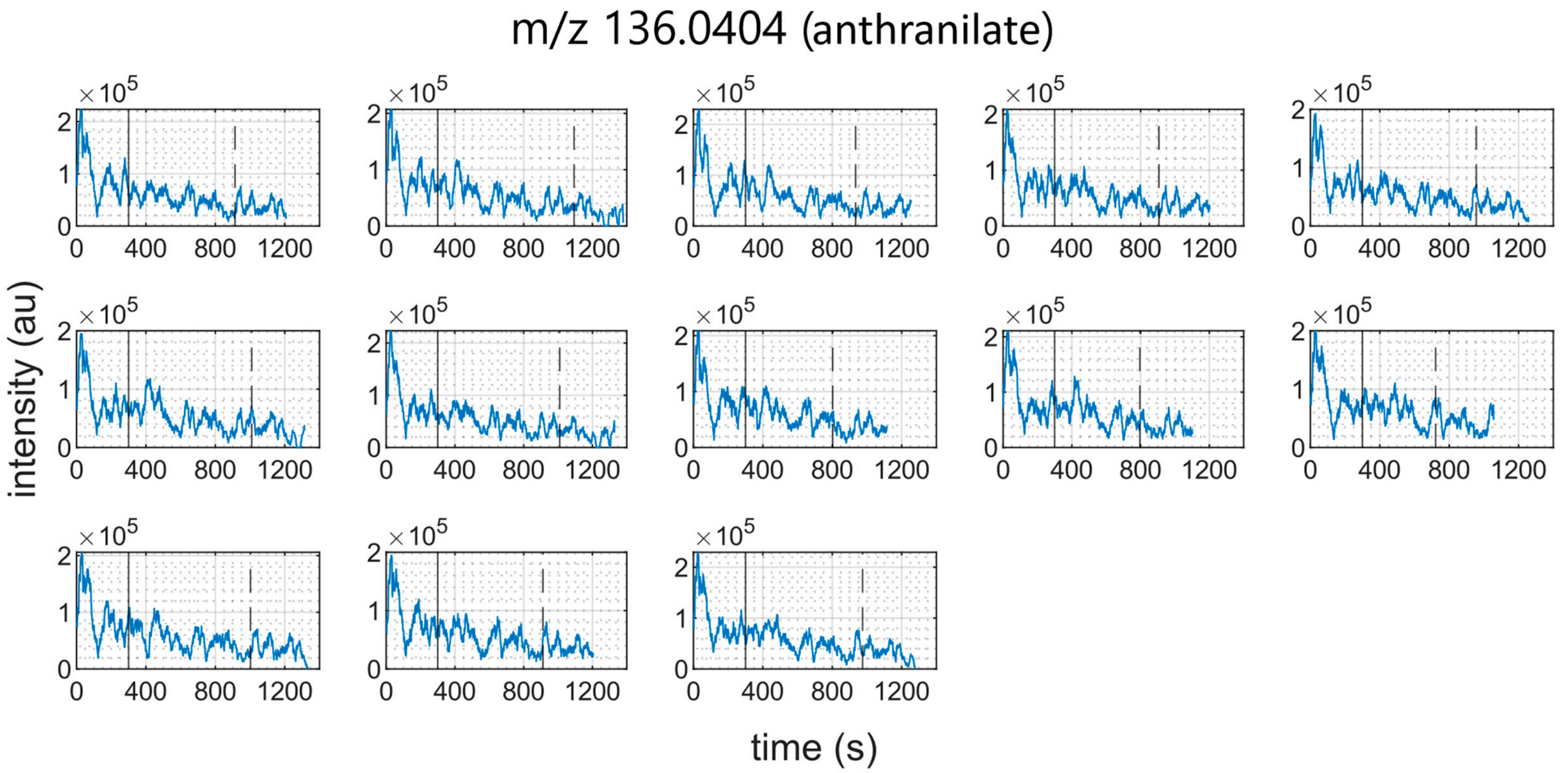
m/z 136.0404 (anthranilate)
intensity (au)
time (s)
×10^5
0
1
2
400
800
1200

m/z 140.0353 (2-aminomuconate semialdehyde)

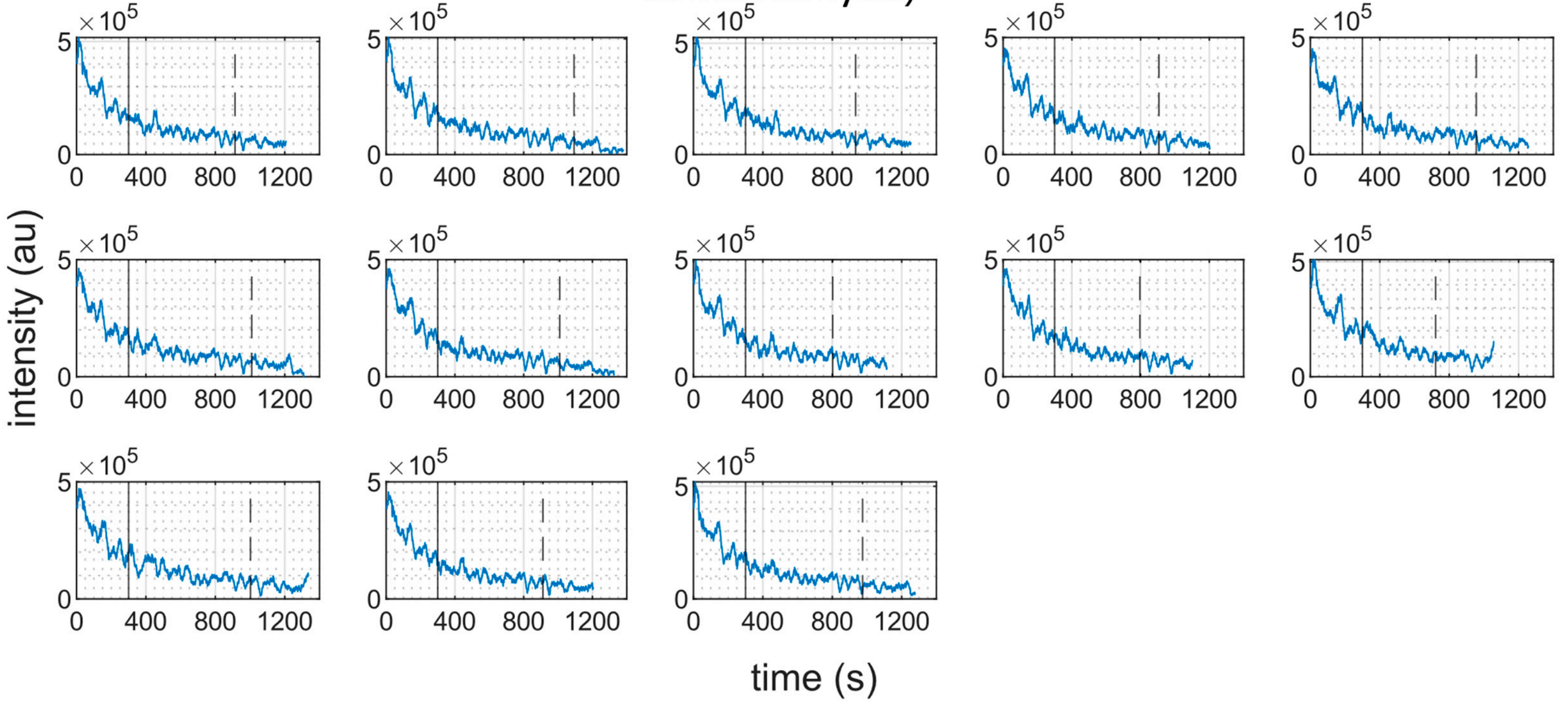

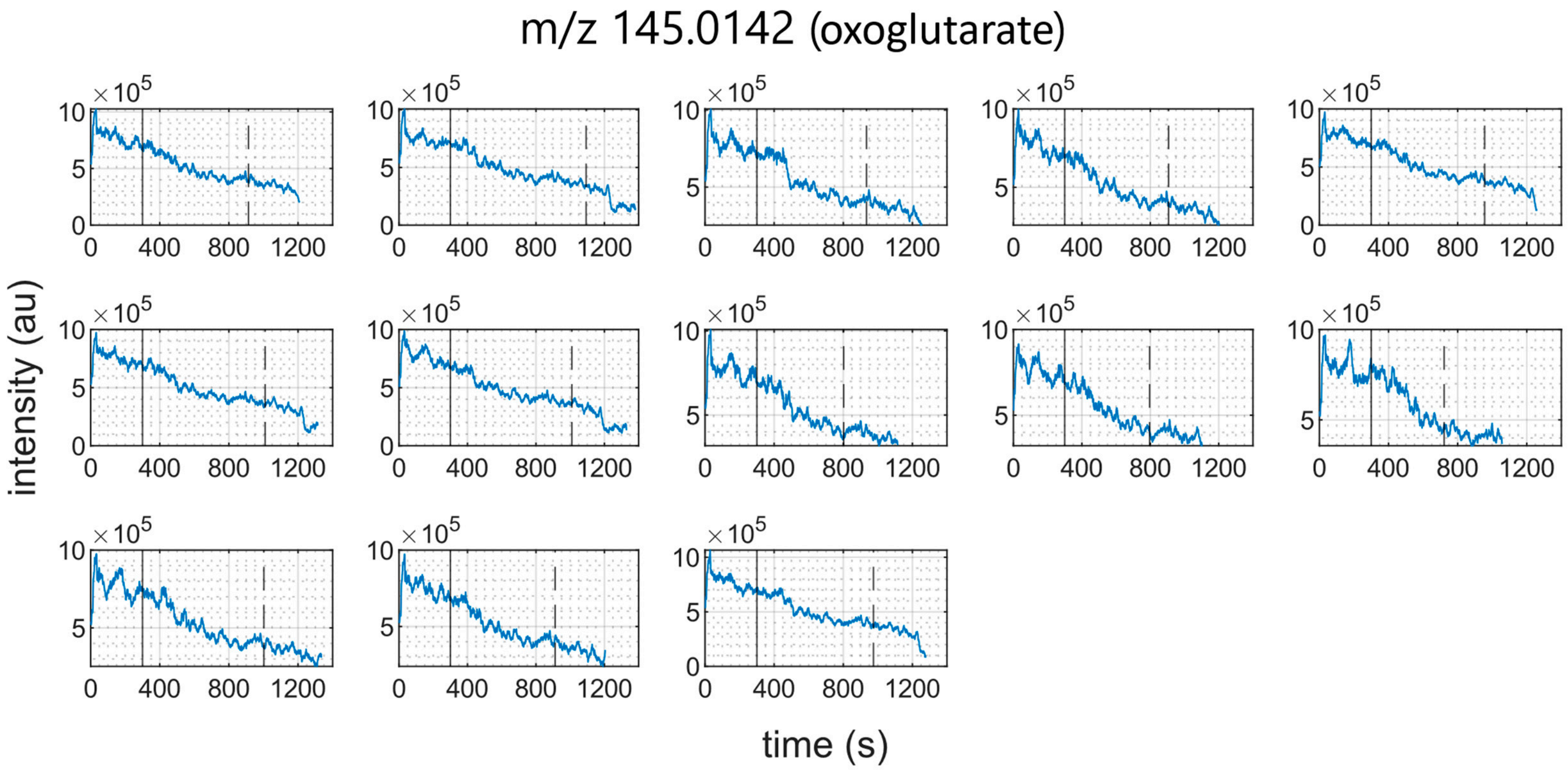
m/z 145.0142 (oxoglutarate)
intensity (au)
time (s)
×10^5
0
400
800
1200
10
5
0

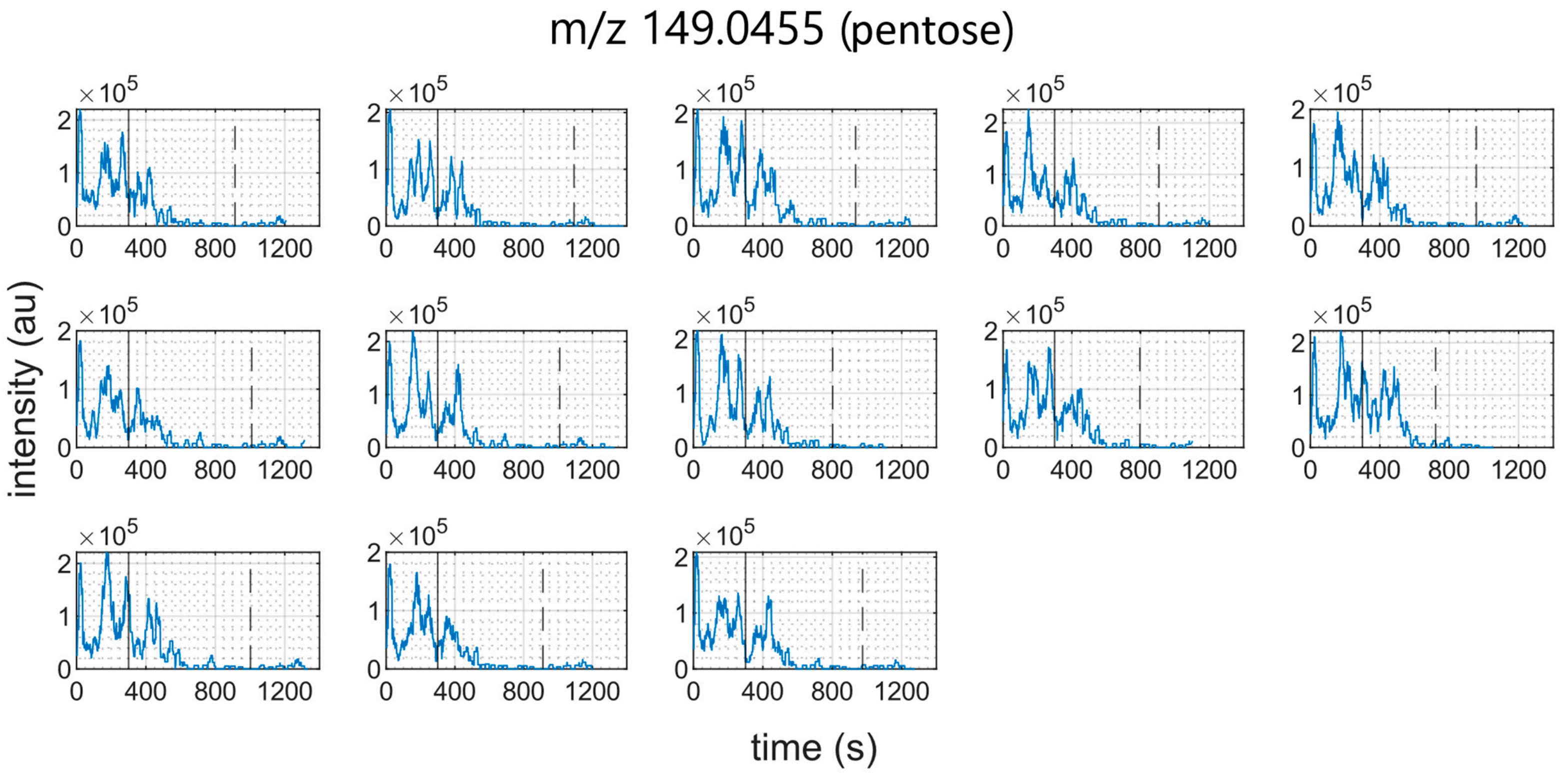
m/z 149.0455 (pentose)
intensity (au)
time (s)
×10^5
0
1
2
0
400
800
1200

m/z 152.0353 (3-hydroxyanthranilate)

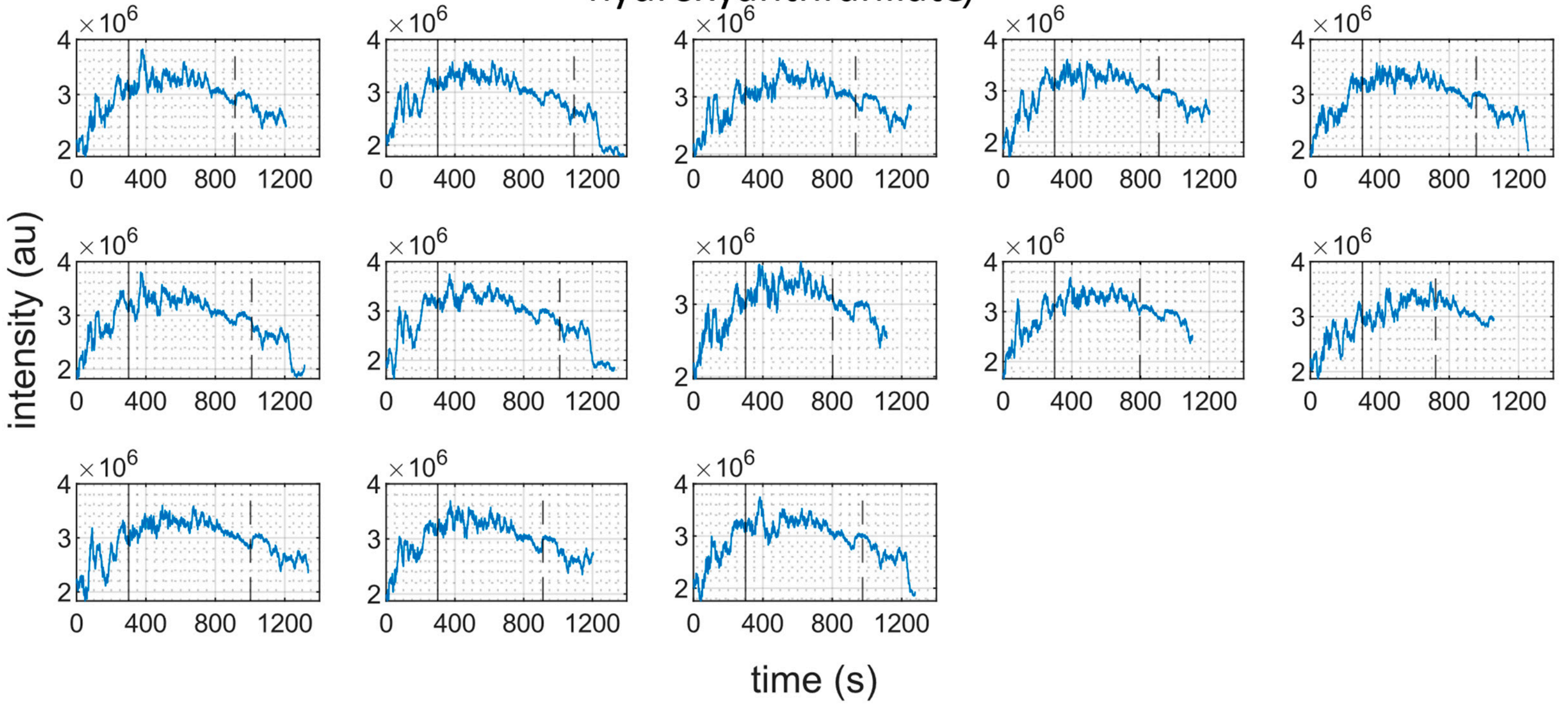

m/z 156.0302 (2-aminomuconate)

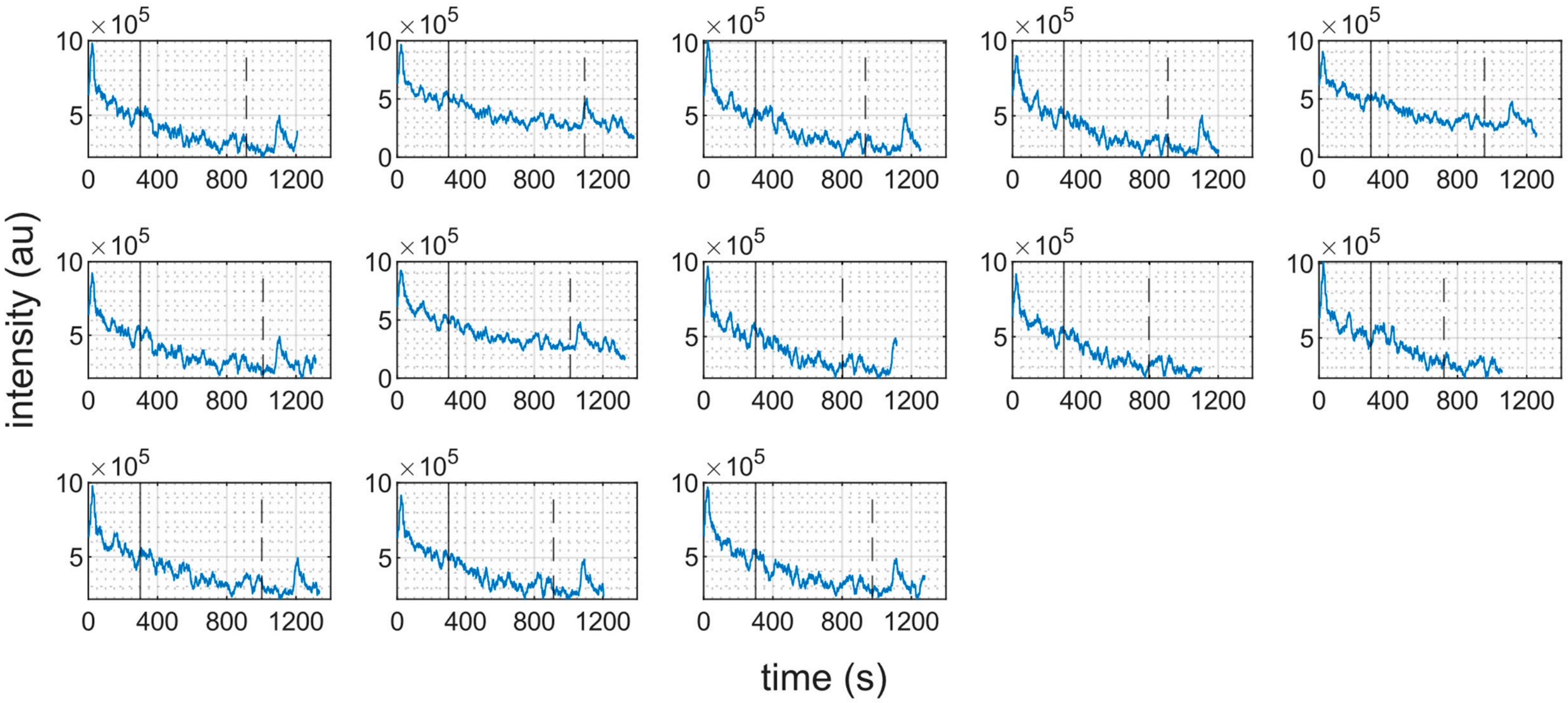

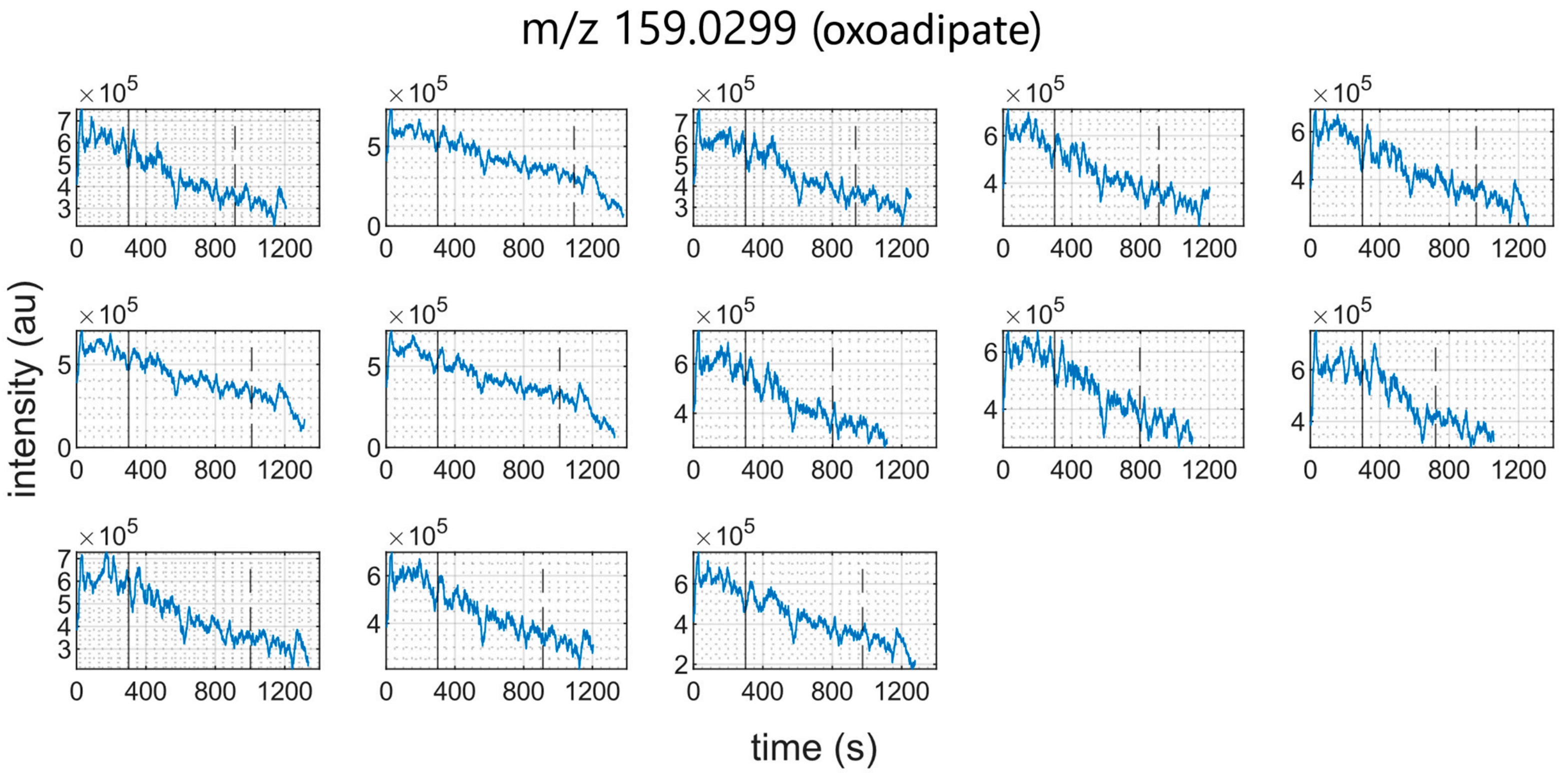
m/z 159.0299 (oxoadipate)
intensity (au)
time (s)

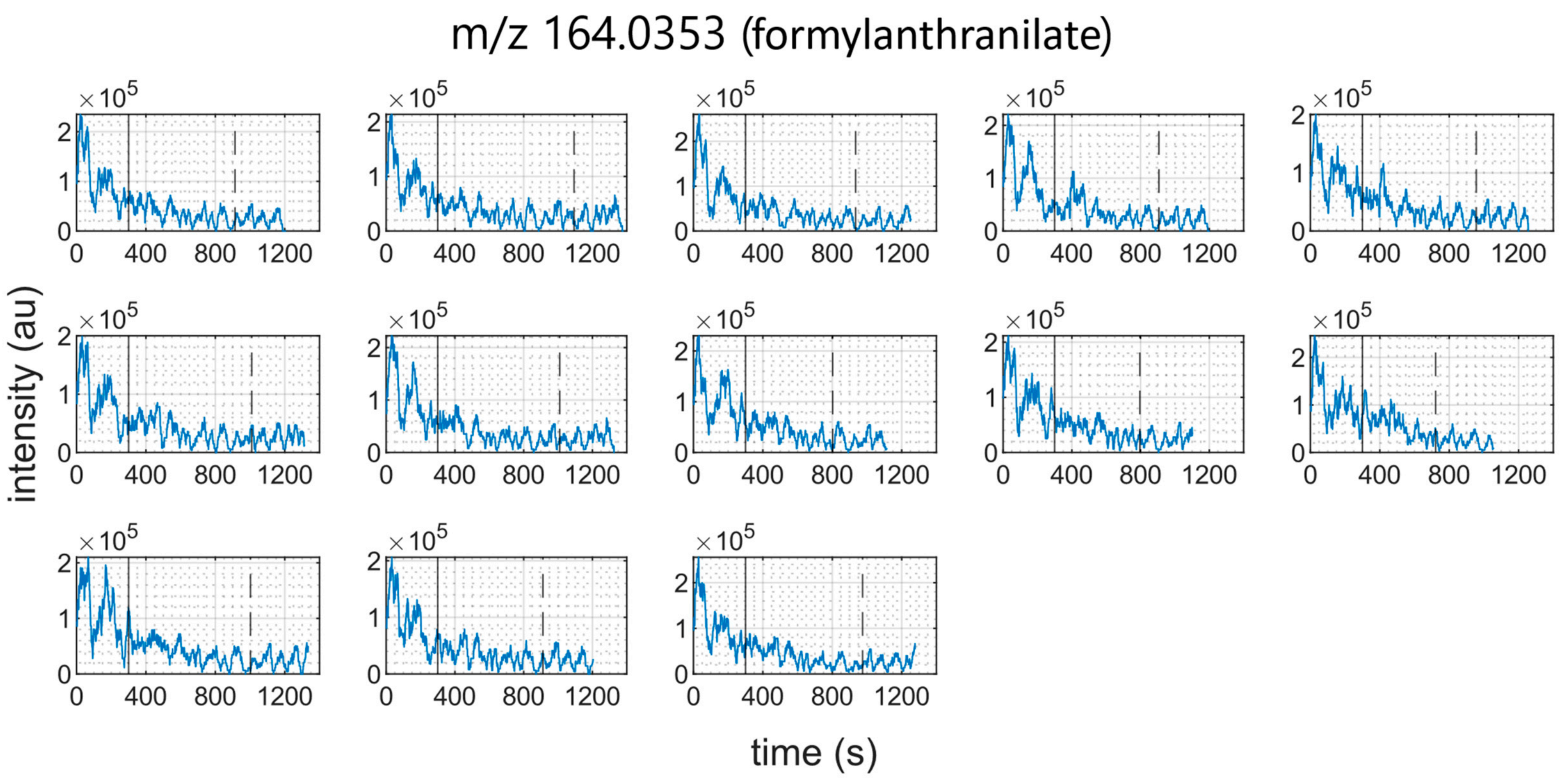
m/z 164.0353 (formylanthranilate)
intensity (au)
time (s)

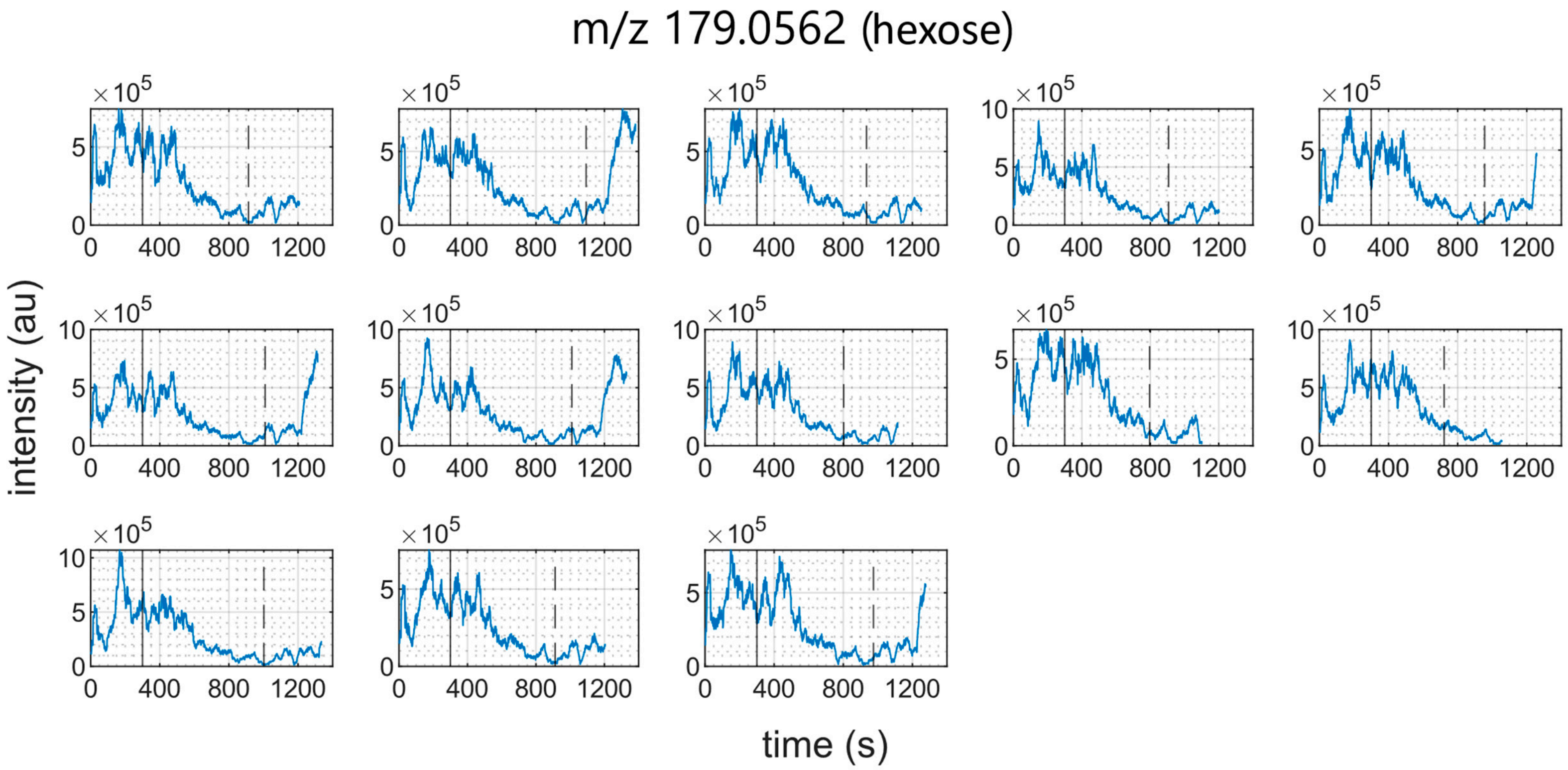
m/z 179.0562 (hexose)
intensity (au)
time (s)

m/z 183.1028 (oxodecenoic acid)

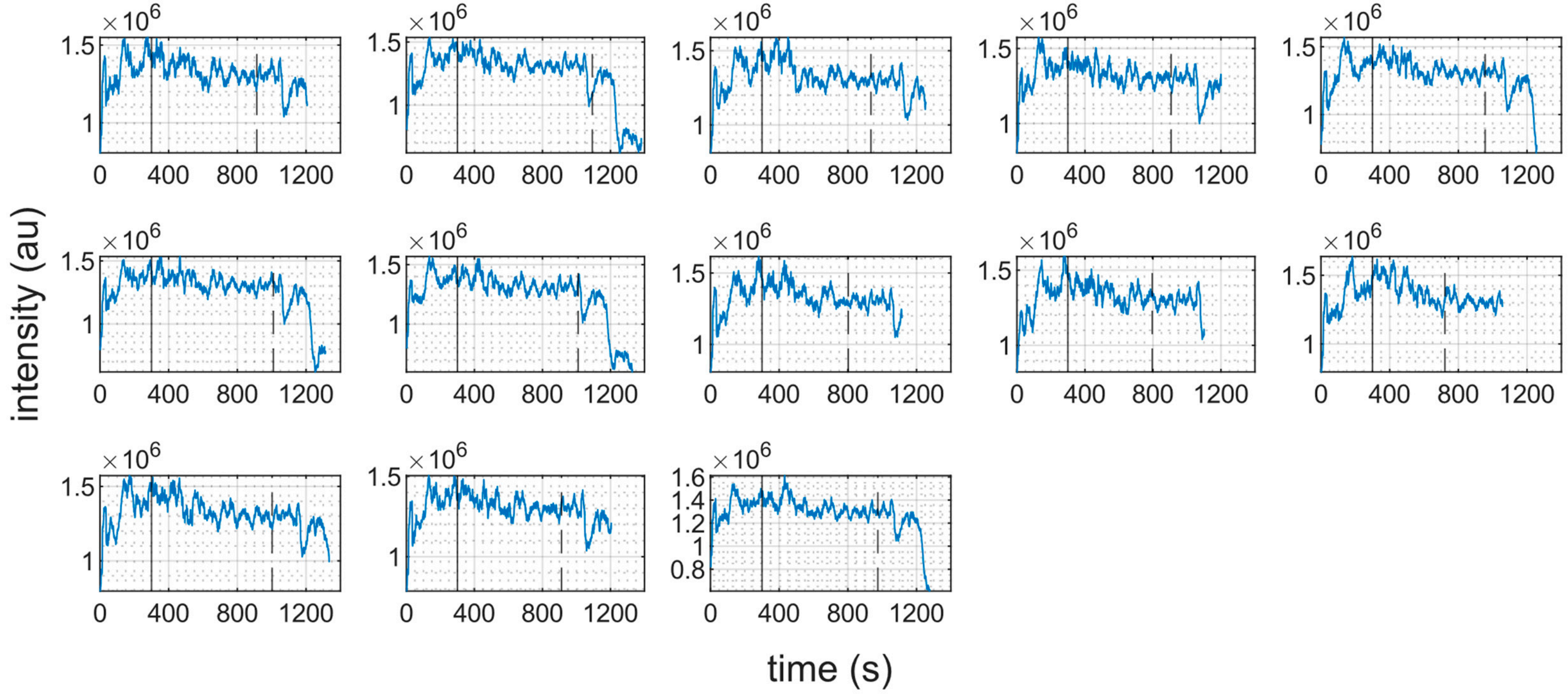

m/z 187.1341 (hyroxydecanoic acid)

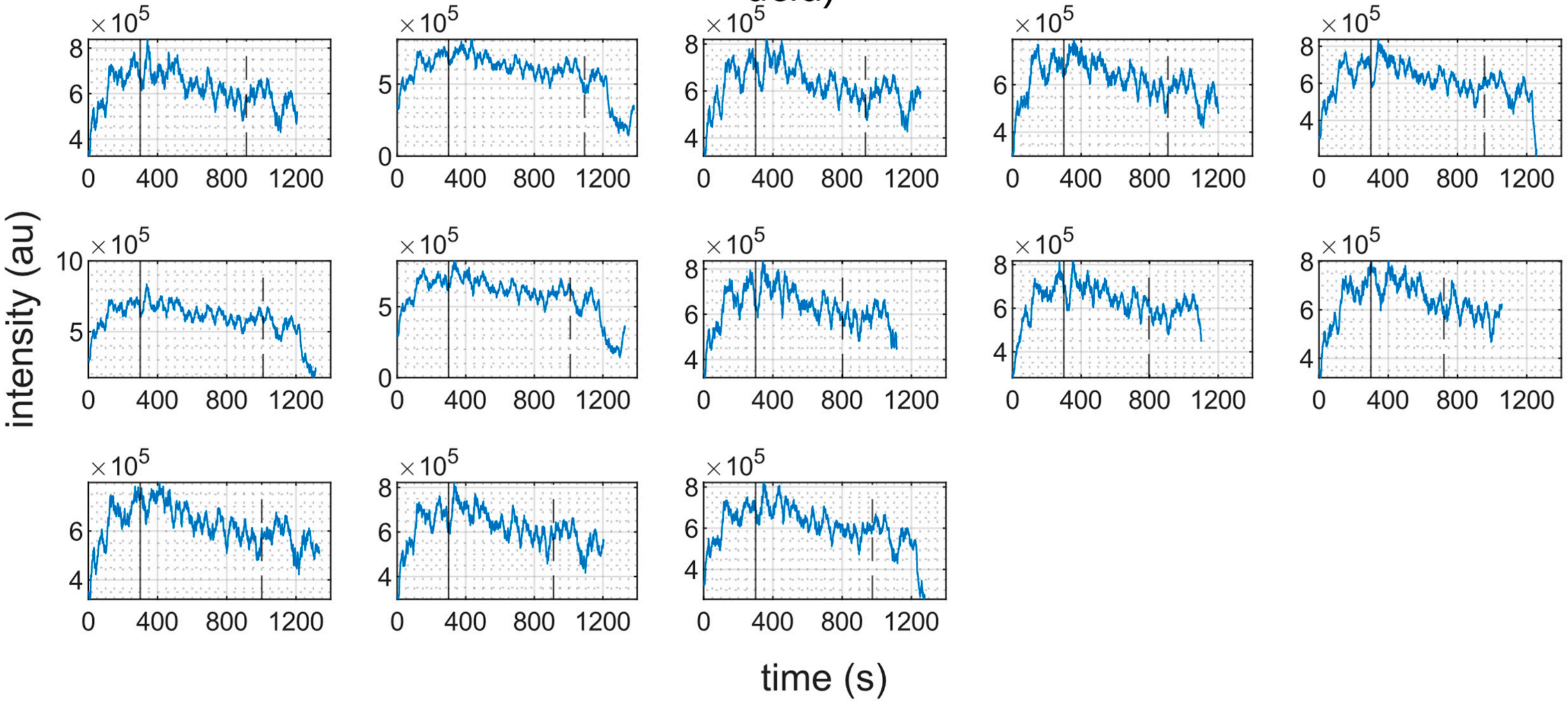

m/z 199.1705 (dodecanoic acid/lauric acid)

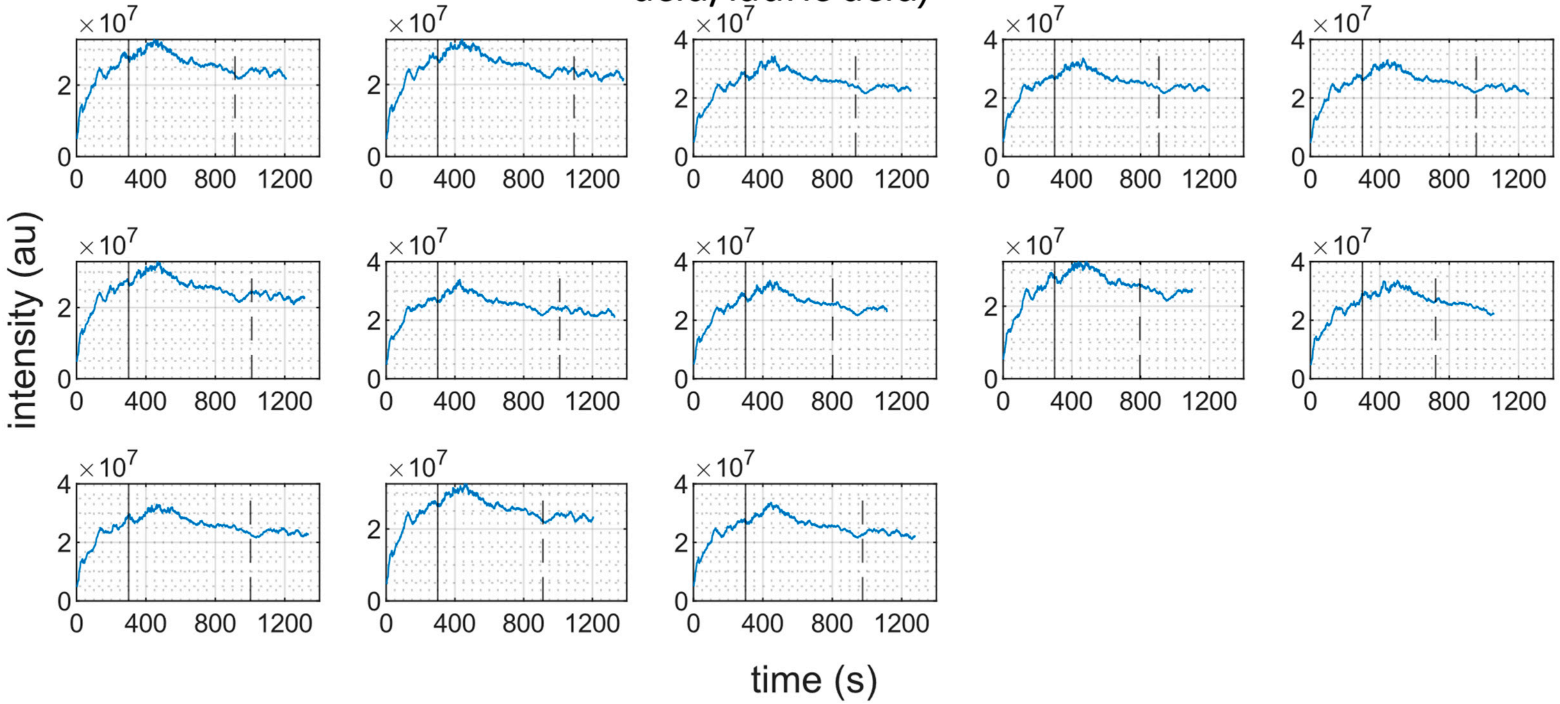

m/z 211.1340 (oxododecenoic acid)

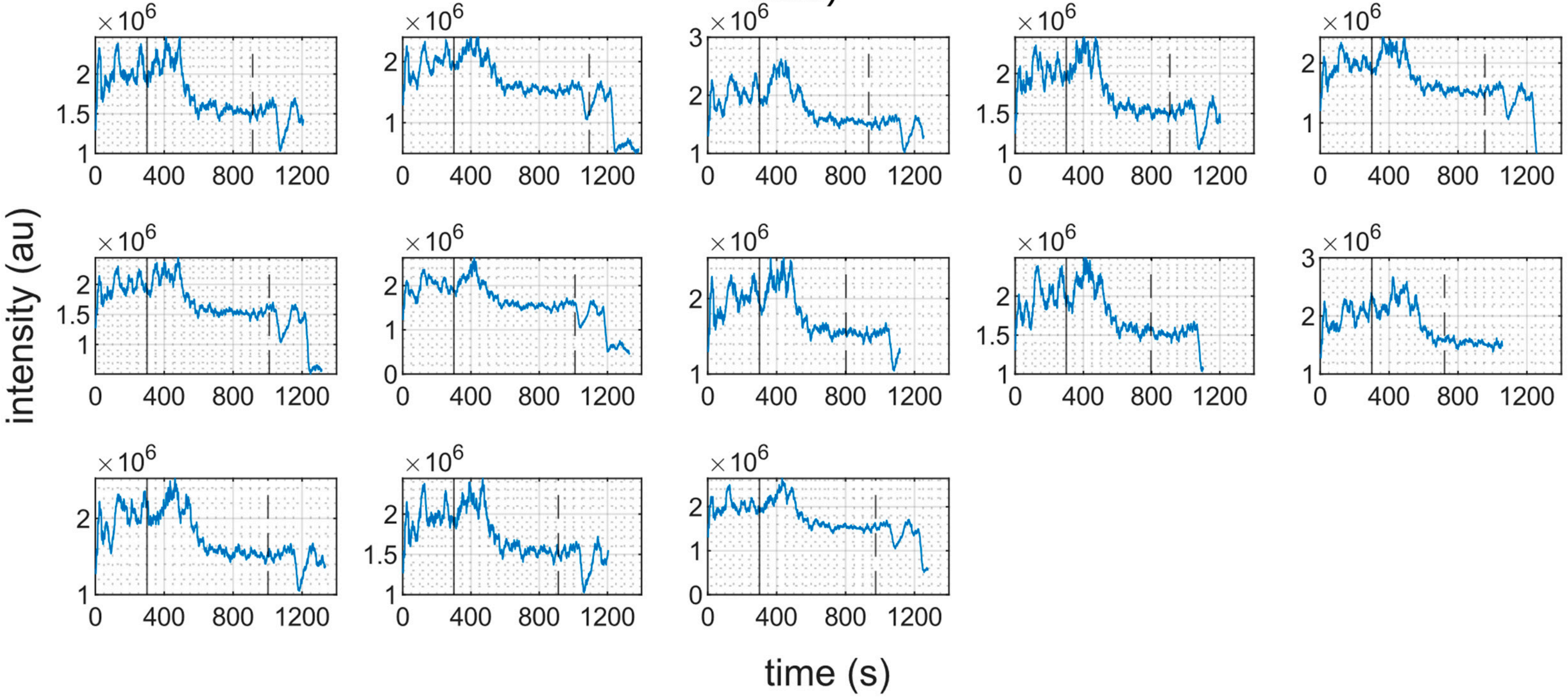

m/z 227.2018 (tetradecanoic acid/myristic acid)

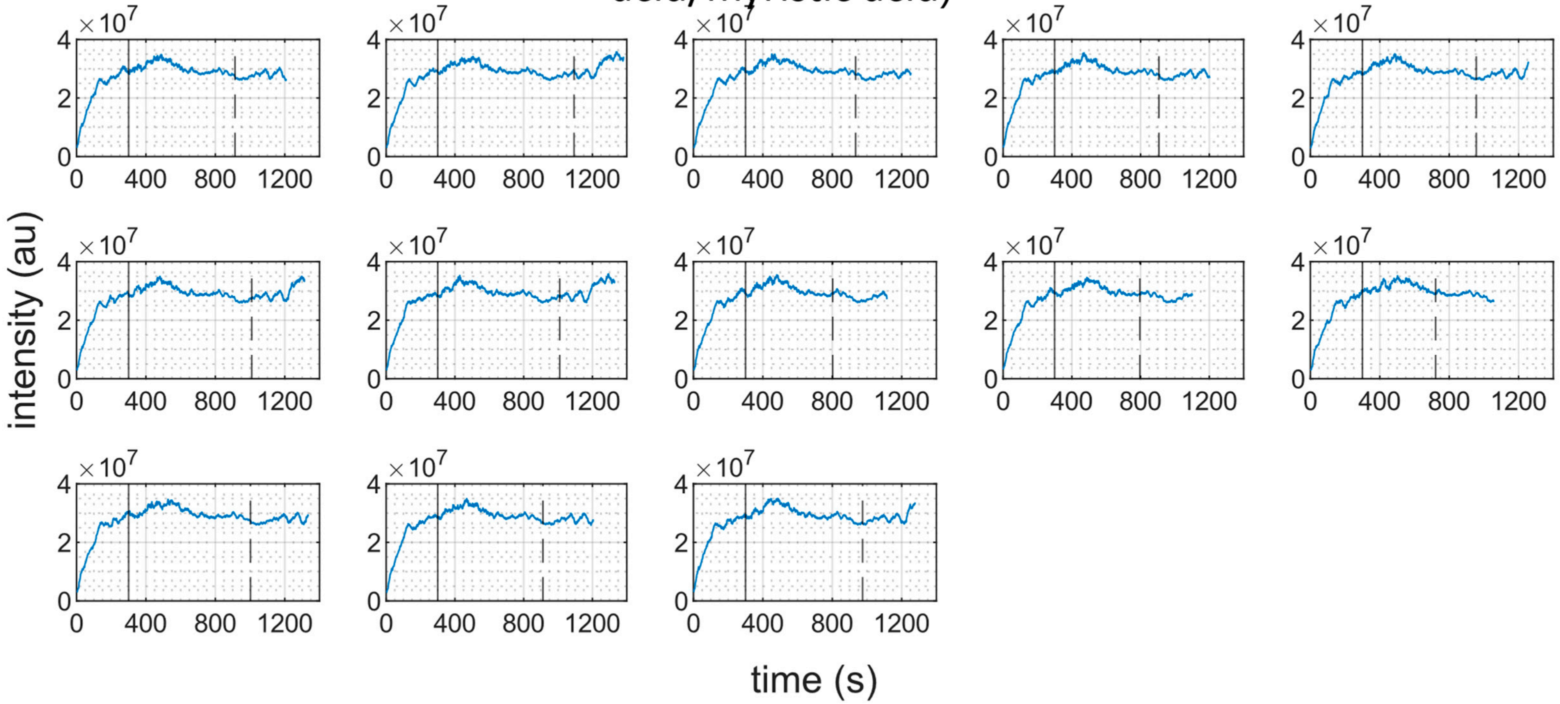

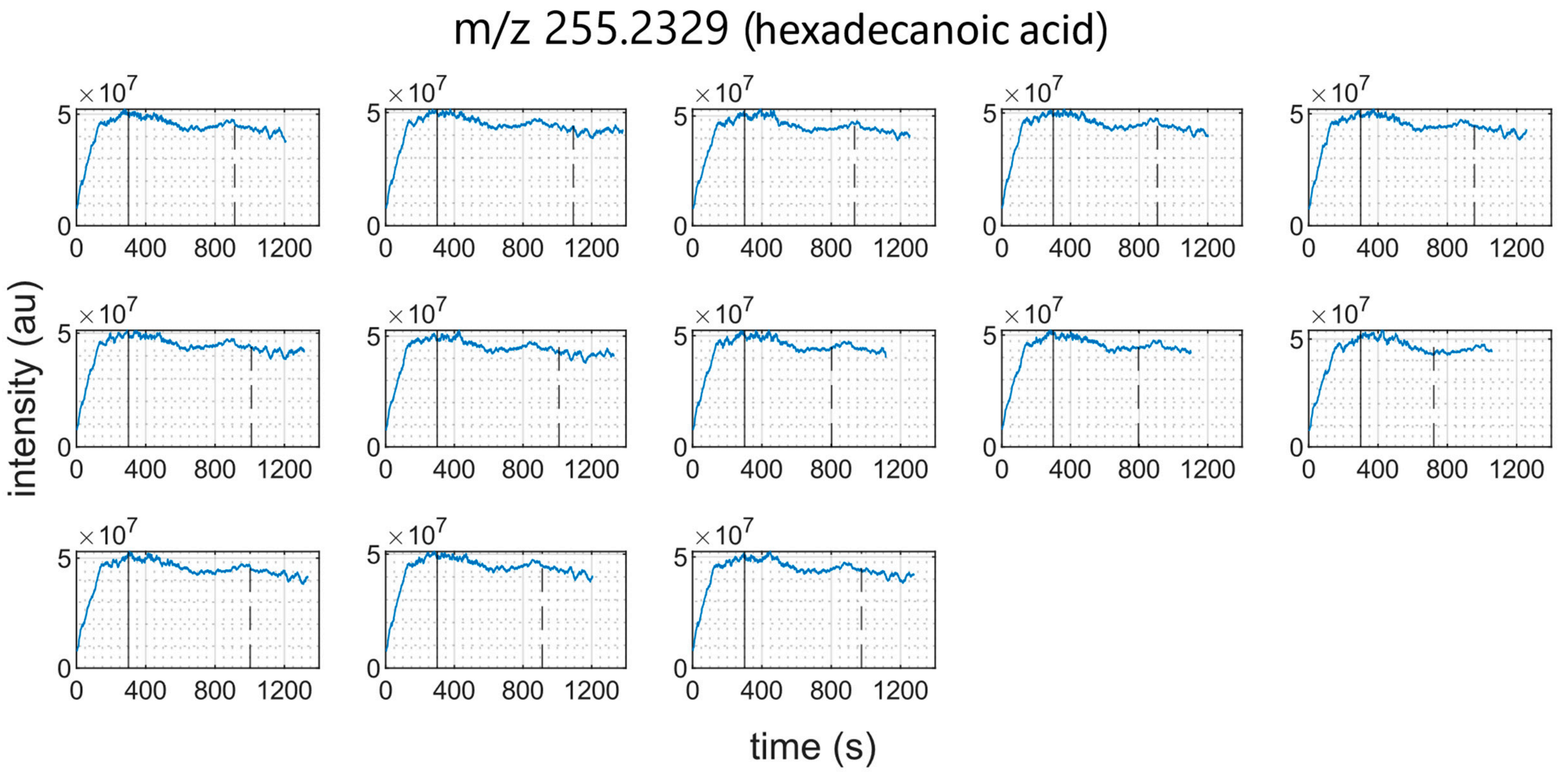

m/z 255.2329 (hexadecanoic acid)
×10^7
5
0
0
400
800
1200
intensity (au)
time (s)

m/z 283.2643 (octadecanoic acid)

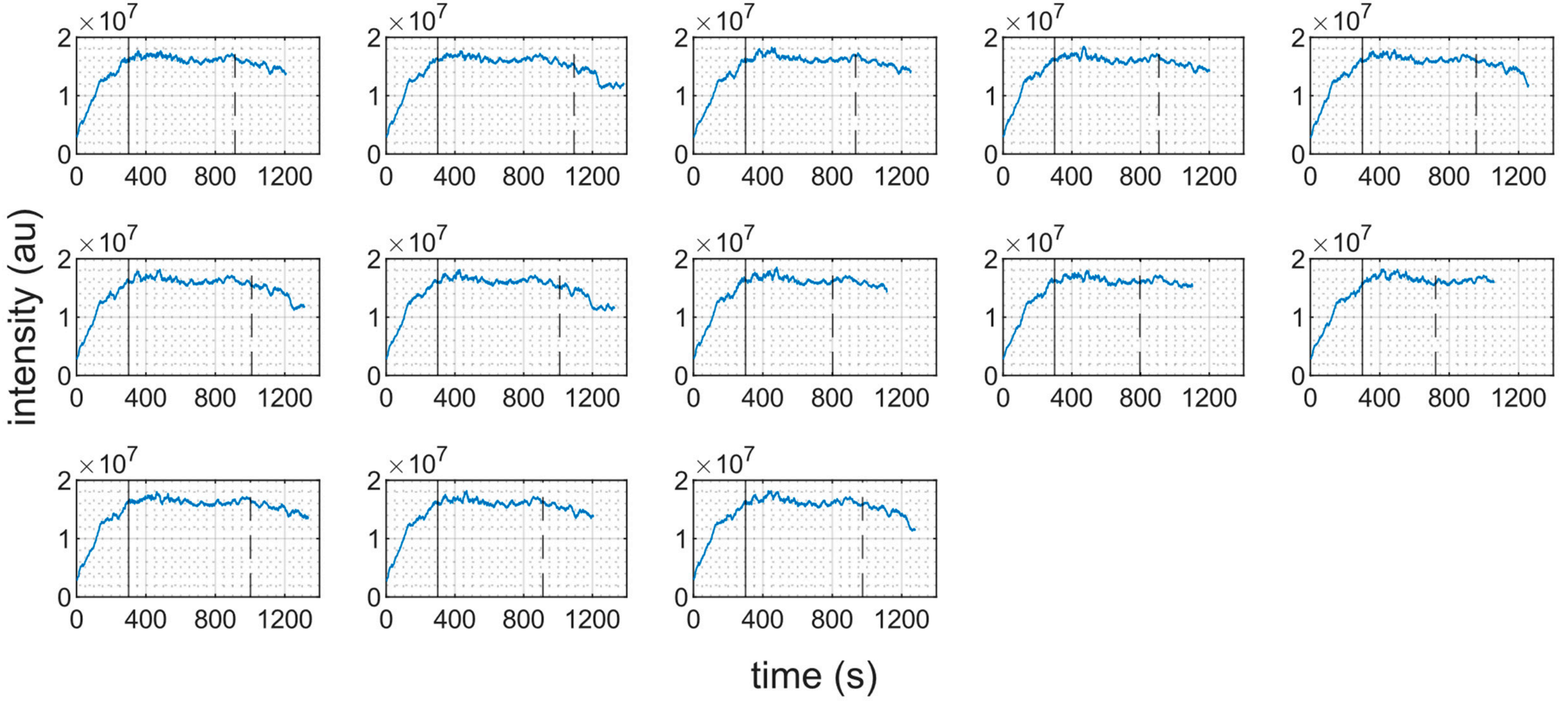

Supplement: Supplementary file 1 [file metabolites-11-00856-s001.zip › Figure_S4.pdf]
